# Temporal dynamics of the friendship paradox in a smartphone communication network

**DOI:** 10.1007/s41109-025-00710-1

**Published:** 2025-05-23

**Authors:** Cheng Wang, Omar Lizardo, David S. Hachen

**Affiliations:** 1 Department of Sociology, Wayne State University, Detroit, MI USA; 2 Department of Sociology, University of California Los Angeles, Los Angeles, CA USA; 3 Department of Sociology, University of Notre Dame, Notre Dame, IN USA

**Keywords:** Friendship paradox, Temporal dynamics, Friendship index, Latent growth-curve model (LGCM), NetHealth project

## Abstract

The friendship paradox, initially discussed by Scott Feld in 1991, highlights a counterintuitive social phenomenon where individuals tend to have fewer friends than their friends do on average. The sociological implications of this paradox are profound, as it can create a distorted understanding of social norms and consequently influence beliefs, attitudes, and behaviors, particularly when highly connected individuals present a skewed representation of those norms. In essence, it can lead individuals to misjudge what is typical or desirable within their social circles. This study investigates the temporal dynamics of the friendship paradox using smartphone communication data from over 600 incoming freshmen at the University of Notre Dame participating in the NetHealth project. By tracking the friendship index– the ratio of an individual’s friends’ average number of friends to their own number of friends– over 119 days during the Fall semester of 2015, we examine how the paradox evolves over time. Our findings reveal that the friendship index stabilizes more rapidly than both the individuals’ own degree and the variation among their friends’ degrees. Results from the latent growth-curve model (LGCM) confirm that while the friendship index continues to increase, its growth rate declines over time. Moreover, the LGCM identifies individual degrees, ethnic backgrounds, and personality traits as influential factors shaping the manifestation and development of the friendship paradox. By exploring the mechanisms underlying this paradox in a dynamic communication network, this study enhances our understanding of the structural factors influencing the evolution of the friendship paradox in digitally mediated interactions.

**Supplementary Information:**

The online version contains supplementary material available at 10.1007/s41109-025-00710-1.

## Introduction

The friendship paradox, first introduced by Feld (1991), refers to the phenomenon where an individual (the “ego”) tends to have fewer friends than the average number of friends their friends (the “alters”) have. Since its introduction, this paradox has been widely observed across various types of networks. For example, a 2012 Pew Research Center survey found that the average Facebook user had 245 friends, while their friends averaged 359, confirming the presence of the friendship paradox on this social media platform (Hampton et al. 2012). Similarly, research by Hodas et al. (2013) revealed that over 98% of Twitter users experience the friendship paradox, as their followers tend to have more followers and followees than they do. Beyond social media, Eom and Jo (2014) demonstrated the paradox’s presence in coauthorship, citation, and publication networks, highlighting its prevalence across a range of social and professional settings.

Researchers have mathematically proven that the friendship paradox exists as long as egos in a network do not share the same degree or number of alters (Just et al. 2015; Cao and Ross 2016; Jackson 2019). Consequently, the friendship paradox frequently appears in the social networks we encounter in everyday life. However, its implications extend far beyond a mathematical curiosity. The paradox can significantly influence our beliefs, attitudes, and behaviors by distorting perceptions of social norms, especially when highly connected individuals exhibit systematically different traits, behaviors, or beliefs (Hodas et al. 2013; Lerman et al. 2016; Jackson 2019). Such distortion can amplify within a network, as a small, highly connected group can exert disproportionate influence. For example, Hodas et al. (2013) demonstrated how the friendship paradox can skew individuals’ perceptions of their friends’ popularity and behaviors, leading to misinterpretations of social norms. Lerman et al. (2016) described the “Majority Illusion,” where highly connected individuals create the false impression that their behaviors are more common, influencing social contagions and shaping network-wide perceptions. Similarly, Jackson (2019) illustrated how the friendship paradox can cause students to overestimate the prevalence of behaviors like drinking or drug use among their peers.

More importantly, investigating the temporal changes of the friendship paradox within a social network, particularly during its early stages, is crucial. Studying how the paradox evolves over time offers valuable insights into how individuals’ perceptions of social norms and behaviors are shaped as the network develops. This is particularly important for understanding how quickly the paradox takes hold and whether its influence intensifies or stabilizes as the network matures. Such knowledge has broader implications for designing interventions that mitigate the distortion of social perceptions, especially in situations where early influences have lasting effects on network dynamics and individual behavior. However, this area remains understudied due to the limited availability of appropriate temporal data.

To address this gap, the present study investigates the temporal dynamics and evolution of the friendship paradox. Specifically, we analyze data from the NetHealth project, a longitudinal study that collected health and social network data from undergraduate students at the University of Notre Dame. Using smartphone communication records from over 600 students spanning a 119-day period, we examine how the friendship paradox develops and evolves over time. Additionally, we explore how personal demographics and psychological traits influence its manifestation and trajectory. Our research aims to answer three key questions: Does the friendship paradox emerge immediately, or does it develop gradually over time? Furthermore, does the friendship index, which measures the degree disparity between egos and alters, change as the network evolves? If so, what patterns and trends define these changes? By addressing these questions, we aim to contribute to a deeper understanding of the friendship paradox and its broader social implications.

## The friendship paradox and its basis

The friendship paradox has been studied through various theoretical lenses, particularly those explaining degree disparities in social networks. While the existence of the friendship paradox is mathematically guaranteed in any network with degree variance (Just et al. 2015; Cao and Ross 2016; Jackson 2019), what remains to be fully explored are the formation processes that create degree disparities among individuals. These processes can increase the gap between the average number of friends an individual has and the number of friends their friends have, thus influencing the magnitude of the observed friendship paradox. The following theories shed light on the mechanisms that drive these degree disparities and contribute to the paradox.

The Pareto Principle, or the 80 − 20 rule (Pareto 1964), offers a foundational explanation for unequal degree distributions in social networks (Barabási 2003; Negreiros et al. 2020). According to this principle, 20% of causes account for 80% of effects, suggesting that a small fraction of individuals (high-degree vertices) dominate network activity. These highly connected individuals (hubs) disproportionately influence the structure of the network by holding far more connections than the majority of individuals. When low-degree individuals connect with these hubs, the degree disparity between individuals and their friends grows, contributing to a larger magnitude of the friendship paradox (Barabási 2003).

Preferential attachment theory, popularized by Barabási and Albert (1999), provides further insight into how networks evolve in a way that amplifies degree disparities and, consequently, the friendship paradox (Pal et al. 2019; Sidorov et al. 2021). In this process, individuals with more connections (high-degree vertices) have a higher probability of attracting new connections. This “rich-get-richer” dynamic (Merton 1968) ensures that popular individuals continue to accumulate connections at a faster rate than others, leading to highly unequal degree distributions. As a result, individuals with fewer connections are more likely to connect with those who are already highly connected, which further increases the magnitude of the friendship paradox as the network grows.

Small world theory (de Sola Pool and Kochen 1978; Watts and Strogatz 1998), exemplified by Stanley Milgram’s “six degrees of separation” experiment (Milgram 1967), provides additional context for understanding the friendship paradox in terms of network structure (Heinrich 2018). Small-world networks combine local clustering with global connectivity. Local clustering ensures that individuals’ friends are likely to know each other, while a few highly connected individuals (hubs) bridge different clusters. This combination of clusters and connectors enhances the visibility of the friendship paradox, as less-connected individuals are more likely to have highly connected friends, widening the degree gap and amplifying the observed paradox.

All of these theories converge on a central concept: degree disassortativity (Newman 2002, 2003), which refers to the tendency of high-degree individuals to connect with low-degree individuals. In real-world social networks, egos may exhibit assortative mixing (preferring alters with similar degrees), disassortative mixing (preferring alters with different degrees), or neutral mixing (displaying no preference for degree similarity or difference) (Newman 2002, 2003). When degree disassortativity is present, this structural property of social networks exacerbates the degree disparity between individuals and their friends, thereby increasing the observed magnitude of the friendship paradox. Conversely, in networks exhibiting degree assortativity, where individuals are more likely to connect with others who have similar degrees, the friendship paradox is less likely to occur. The friendship paradox underscores the inherent structural inequalities within social networks, where a small group of highly connected individuals can disproportionately influence the overall network. Understanding these dynamics is crucial for identifying the processes that drive the increasing disparity in network degree distributions and for exploring the broader implications for social interactions, information diffusion, and network behavior.

By exploring these theories, researchers can better understand not just the existence of the friendship paradox, but also the underlying mechanisms that magnify it, offering insights into the structural patterns that shape social networks.

## The sociology of the friendship index

From a sociological perspective, understanding the friendship paradox requires examining not just its mathematical foundation but also its broader social implications. While the paradox is well-documented mathematically, its real-world consequences– such as misperceptions of norms or exaggerated estimations of adverse behaviors– are not automatic. Instead, these effects emerge through a complex interplay of social, structural, and individual factors that shape how the paradox manifests in everyday life.

For instance, the likelihood of misperceptions depends on how frequently individuals interact with highly connected peers. These individuals, often the most active in communication and content creation, exert a disproportionate influence in shaping others’ perceptions (Hodas et al. 2013). Beyond individual influence, the structural properties of networks– including clustering, modularity, and bridging ties– play a crucial role in determining how information spreads and interacts with the friendship paradox. Clustering, for example, can reinforce echo chambers that distort perceived norms (Lerman et al. 2016), while bridges between network segments can accelerate the diffusion of behaviors or beliefs (Jackson 2019).

In addition to these structural factors, demographic and psychological characteristics influence how individuals experience the friendship paradox. Variations in social connections based on gender and race/ethnicity may either amplify or mitigate its effects across different populations. Men, for instance, tend to maintain broader social networks compared to women, leading to differences in degree centrality and perceptions of connectedness (Feld 1991; McPherson et al. 2006). Similarly, racial and ethnic minorities often navigate a balance between in-group and out-group ties, reflecting cultural norms that emphasize community and solidarity. This dynamic, as highlighted by Blau (1977), creates significant heterogeneity in social interactions, potentially intensifying disparities in perceived popularity or influence.

Beyond demographic factors, psychological traits further shape how individuals interpret and respond to network discrepancies. Those with higher extraversion and lower neuroticism tend to exhibit greater degree centrality, making their social connections more visible (Kalish and Robins 2006). Likewise, individuals with high self-esteem, despite maintaining larger social networks and receiving more support, may still perceive intensified disparities if their peers appear even more socially connected (Marshall et al. 2014). These psychological dimensions underscore the importance of considering individual-level differences when analyzing the friendship paradox within social networks.

Sociologists are not only concerned with static social patterns but also with how these patterns evolve over time. The friendship paradox is no exception– while much of the existing research treats it as a fixed condition, exploring its temporal dynamics provides deeper insight into its influence on network behavior and social perception (Eom and Jo 2014; Lerman et al. 2016). Examining how the paradox develops over time can reveal how quickly individuals’ perceptions of social norms become distorted as networks form and evolve (Hodas et al. 2013; Jackson 2019). This is particularly relevant in settings such as schools, workplaces, and online communities, where early social interactions play a crucial role in shaping long-term group behavior and decision-making (Feld 1991; Eom and Jo 2014). Additionally, understanding whether the paradox intensifies or stabilizes over time can help researchers pinpoint the conditions under which its effects on peer influence, social contagions, and behavioral norms become most pronounced (Lerman et al. 2016; Jackson 2019).

Investigating these temporal dynamics also carries practical implications for mitigating the paradox’s effects. In public health campaigns and educational initiatives, addressing early misperceptions of social norms could prevent harmful behaviors from spreading through networks (Hodas et al. 2013; Eom and Jo 2014; Lerman et al. 2016). Similarly, insights into the evolution of the friendship paradox could inform strategies to counteract biases in social media algorithms, which often amplify the voices of highly connected individuals and distort perceptions of popularity and social influence (Lerman et al. 2016; Jackson 2019). The current study directly contributes to this growing body of research by examining how the friendship paradox unfolds over time using a high-resolution dataset of smartphone communication networks. By analyzing the dynamic patterns of the friendship index across multiple demographic and psychological factors, this study offers empirical insights into the stability, variability, and potential mitigation of the paradox in real-world social interactions.

## Notations for studying friendship paradox

To further illustrate the concepts and theories, let’s begin with a simple undirected network graph *G*(*V*, *E*), where *V* represents the set of vertices (i.e., individuals in the network) and *E* represents the set of edges (i.e., connections between individuals in the network). In this example, the network consists of four vertices: Aaron, Beatrice, Chris, and David. Aaron is connected to Beatrice; Beatrice is connected to Aaron, Chris, and David; Chris is connected to Beatrice and David; and David is connected to both Beatrice and Chris.

By examining the connections, we can easily determine the degree ($\:{d}_{i})\:$of each vertex in the graph, as shown in Table 1. Aaron’s degree is 1 since he is only connected to Beatrice. Beatrice, on the other hand, has a degree of 3, making her the most popular vertex in this network. Both Chris and David have a degree of 2, respectively.

**Table 1:** Summary of network statistics for an example network graph, detailing nodal degree, total degree of alters, and the average degree of alters

Ego	$\:{d}_{i}$	$\:{\sum\:}_{j\in\:V}{d}_{j}$	$\overline{{d_{j} }} = \mathop \sum \nolimits_{{j \in V}} d_{j} /d_{i}$
Aaron	1	3	3
Beatrice	3	5 = 1 + 2 + 2	1.67
Chris	2	5 = 3 + 2	2.50
David	2	5 = 3 + 2	2.50
Mean	2		2.25

To gain further insight, we can calculate the cumulative degree of each vertex’s alters. For Aaron, this sum is 3, as all connections are solely with Beatrice. Beatrice’s cumulative degree is 5, which is the sum of 1 (from Aaron) plus 2 (from Chris) plus 2 (from David). Similarly, both Chris and David have a cumulative degree of 5, representing the sum of 3 (from Beatrice) and 2 (from the other individual). Consequently, the average degree of each vertex’s alters is 3 for Aaron, 1.67 for Beatrice, and 2.5 for both Chris and David.

In this particular case, the friendship paradox becomes apparent, as three out of the four vertices in the network experience it. For the entire network, the mean of each vertex’s degree ($\:{d}_{i}$) is 2, while the mean of the average degree of each vertex’s alters ($\overline{{d_{j} }} = \mathop \sum \nolimits_{{j \in V}} d_{j} /d_{i}$) is 2.25. Notably, the latter value is greater than the former, providing clear evidence of the presence of the friendship paradox.

Using the parameters presented in Table 1, Pal and colleagues (2019) introduced a novel metric called the friendship index. This index measures the ratio of the average degree of an individual’s alters to their own degree within the network. The friendship index serves as a local network indicator, as each vertex, or ego, has their own specific value for this index. In Eq. (1), the friendship index, denoted as $\:{FI}_{i}$, is calculated by dividing the average degree of the ego’s alters ($\bar{d}_{j}$) by the ego’s degree ($\:{d}_{i}$). Furthermore, the numerator can be alternatively expressed as the sum of the degrees of a vertex’s alters over the vertex’s degree. This formulation allows for the conversion of the friendship index into the ratio of the sum of the degrees of a vertex’s alters to the square of the vertex’s degree.

1 $$FI_{i} = \frac{{\overline{{d_{j} }} }}{{d_{i} }} = \frac{{\mathop \sum \nolimits_{{j \in V}} d_{j} /d_{i} }}{{d_{i} }} = \frac{{\mathop \sum \nolimits_{{j \in V}} d_{j} }}{{d_{i}^{2} }}$$

Previous research (Pal et al. 2019) has primarily treated the friendship index as a static indicator, offering a snapshot of the degree disparity between an ego and their alters at a single point in time. In contrast, this study builds on prior work by extending the friendship index into a dynamic metric, denoted as $FI_{{it}} = ~\frac{{\overline{{d_{{jt}} }} }}{{d_{{it}} }} = \frac{{\mathop \sum \nolimits_{{j_{t} \in V_{t} }} d_{{jt}} }}{{d_{{it}}^{2} }}$, representing the friendship index of a vertex *i* at time *t*. This temporal adaptation allows us to track how the disparity between a vertex’s degree and the degrees of its alters shifts over time as the network evolves. By analyzing these temporal dynamics, we can observe whether the degree disparity intensifies or stabilizes as the network matures, offering new insights into the evolving nature of the friendship paradox. This dynamic approach enables us to address a significant gap in the literature, providing a more nuanced understanding of how the friendship paradox develops and changes as the network grows and social interactions fluctuate.

## Materials and methods

### Data

The NetHealth dataset, which is approved by the Institutional Review Boards (IRB) at the University of Notre Dame and publicly available at its official website (http://sites.nd.edu/nethealth/data-2/), consisting of data collected from 692 undergraduate students attending the University of Notre Dame, is used in this study. The data was collected from August 2015, when the students started their undergraduate program, until May 2019, when they graduated.

The NetHealth project collected three types of metadata from participants. Firstly, over 62 GB of Fitbit data were gathered, providing minute-by-minute information on heart rate, physical activity, and sleep patterns. Secondly, participants installed a smartphone app developed by the project team, which synchronized communication data to a secure server. This process captured more than 60 million communication records from and to the 692 students, including details on calls and messages. Finally, eight waves of surveys were administered each semester via the Qualtrics platform between August 2015 and May 2019. These surveys collected information on students’ behaviors, attitudes, opinions, and physical and psychological states. Using a name generator, participants listed up to 20 individuals with whom they spent time communicating or interacting with, such as family members, romantic partners, friends, acquaintances, neighbors, co-workers, or members of the same organization. For each listed alter, participants provided demographic and relational details, including contact information, age, sex, race/ethnicity, educational attainment, religious preference, relationship length, perceived closeness, similarity, and frequency of interactions over the past three months.

For this study, we specifically analyzed smartphone communication records collected through smartphone app. These records include details on the caller, the callee, call type (such as voice call, text message, or multimedia message; emails were not captured), communication start and end times, and the duration of voice calls or length of text messages. It is important to note that the focus of this study is on the communication events that occurred exclusively between the 692 participants included in the NetHealth dataset.

The study period spans the Fall semester of 2015, from August 23 to December 19, 2015, covering a total of 17 weeks, or 119 days. To calculate the friendship index $\:{FI}_{it}$, undirected networks were constructed daily based on smartphone interactions among the 692 students throughout the 119 days. A total of 9,732 unique ties were recorded among participants during this period.

Notably, the smartphone communication network closely aligned with the self-reported friendship network, with 94% of the friendships reported in the Qualtrics surveys also appearing in the communication network data used for this study. However, the reverse is not true– only 25% of the 9,732 phone interaction ties among the participants were reported in the surveys. This discrepancy likely arises from the survey design, which limited participants to listing up to 20 individuals, often prioritizing family members, close friends, and acquaintances outside the NetHealth participant network. Consequently, more casual or short-term interactions with other participants were underrepresented in the survey data. This distinction highlights the complementary strengths of the two data sources: while the surveys provide static snapshots of relationships months apart, likely emphasizing a selective subset of stronger or more stable ties, the smartphone communication data capture continuous, high-frequency interactions, offering a more granular and dynamic view of social connections. The latter enables a more detailed examination of evolving social patterns, such as changes in the friendship index over time.

Table 2 presents the sample characteristics of controlled variables, including personal demographics and psychological traits, collected via the entry survey at the start of the project. The sample demonstrates a balanced representation of genders, with slightly more than half of the participants being female. In terms of racial composition, the majority of participants identified as White, while approximately 13% were Latinos, 6% were Black Americans, 9% were Asian Americans, and 7% were foreign students. Regarding religious affiliation, around 73% of the NetHealth participants identified as Catholics, while approximately 10% were Protestants, 4% were affiliated with other religions, and 12% reported no religious affiliation.

**Table 2:** Descriptive statistics for study participants, including demographic variables (gender, race/ethnicity, religious preference), personality traits, and psychological characteristics

Variables for each individual	Mean (SD) or *n* (%)
Female (1 = yes)	359 (51.88%)
Ethnoracial category	
White (1 = yes)	451 (65.17%)
Latino (1 = yes)	88 (12.72%)
Black American (1 = yes)	41 (5.92%)
Asian American (1 = yes)	61 (8.82%)
Foreigner (1 = yes)	51 (7.37%)
Religious preference	
Catholic	505 (72.98%)
Protestant	71 (10.26%)
Other religion	30 (4.34%)
No religion	86 (12.43%)
Extraversion	0.00 (0.72)
Agreeableness	0.00 (0.62)
Conscientiousness	0.00 (0.65)
Neuroticism	0.00 (0.66)
Openness	0.00 (0.59)
Self-esteem	0.00 (0.72)
Loneliness	0.00 (0.57)
Self-regulation	0.00 (0.61)
Depression	0.00 (0.54)
Number of individuals	692 (100.00%)

We included from the entry survey ratings for the Big Five personality factors, namely extraversion (derived from 8 items, Cronbach’s α = 0.87), agreeableness (derived from 9 items, Cronbach’s α = 0.79), conscientiousness (derived from 9 items, Cronbach’s α = 0.83), neuroticism (derived from 8 items, Cronbach’s α = 0.82), and openness (derived from 10 items, Cronbach’s α = 0.79), following the framework proposed by John et al. (1991). Additionally, we assessed other psychological characteristics from the entry survey, including self-esteem (derived from 10 items, Cronbach’s α = 0.90; Rosenberg 1965), loneliness (measured using 15 items from the Social and Emotional Loneliness Scale for Adults [SELSA], Cronbach’s α = 0.85; DiTommaso and Spinner 1993), self-regulation (measured using 12 items from the Self-Regulation Questionnaire [SRQ], Cronbach’s α = 0.84; Brown et al. 1999), and depression (measured using 20 items from the Center for Epidemiologic Studies Depression Scale [CES-D], Cronbach’s α = 0.88; Radloff 1977).

## Active, cumulative, versus effective degree

**Fig. 1:**
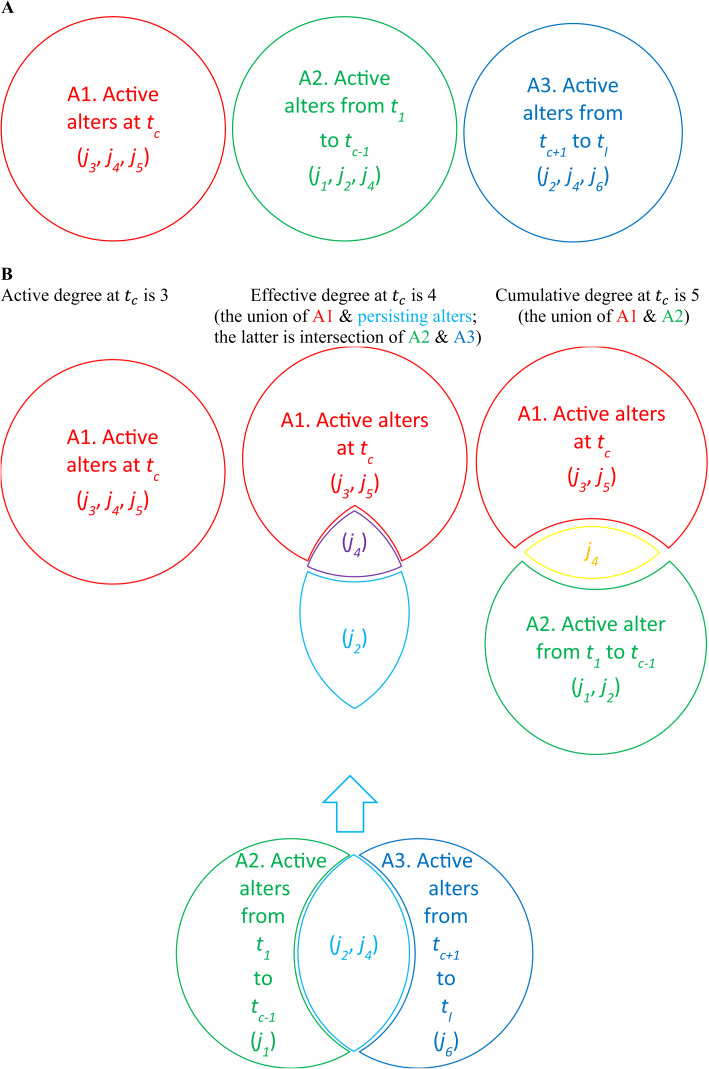
Definition of active degree (number of alters at a specific time point), effective degree (a combination of active and persisting alters to capture stable connections), and cumulative degree (total number of alters over time)

Before delving into our research questions, it is crucial to clarify how to measure degree (*d*_*it*_) in this study, as different approaches yield varying insights into the temporal dynamics of ego networks. To aid in understanding, we have provided a visual representation in Fig. 1, accompanied by detailed textual explanations.

One common approach is to calculate the *active degree*, which counts the number of alters an ego has at the current time point (*t*_*c*_). For example, as shown in Fig. 1A (left) and Fig. 1B (left), at *t*_*c*_, the ego *i* has three active alters: {*j*_*3*_, *j*_*4*_, *j*_*5*_}, resulting in an active degree of 3. While straightforward, this method has limitations. For instance, ties that are only active intermittently, such as calls to parents on weekends, may disappear from the ego network on weekdays, potentially leading to misleading conclusions when using day-level active degrees as the sole measure.

Alternatively, the *cumulative degree* considers the total number of alters an ego has interacted with from the first time point (*t*_*1*_) to the current time point (*t*_*c*_). As depicted in Fig. 1A (center), in addition to the three active alters {*j*_*3*_, *j*_*4*_, *j*_*5*_} at *t*_*c*_, the ego *i* has interacted with three alters {*j*_*1*_, *j*_*2*_, *j*_*4*_} between *t*_*1*_ and *t*_*c*−1_, the time point before *t*_*c*_. In Fig. 1B (right), the cumulative active alters are the union of {*j*_*1*_, *j*_*2*_, *j*_*4*_} and {*j*_*3*_, *j*_*4*_, *j*_*5*_}, resulting in {*j*_*1*_, *j*_*2*_, *j*_*3*_, *j*_*4*_, *j*_*5*_}. Among these, *j*_*4*_ is active both at *t*_*c*_ and between *t*_*1*_ and *t*_*c*−1_, *j*_*1*_ and *j*_*2*_ are only active between *t*_*1*_ and *t*_*c*−1_, and *j*_*3*_ and *j*_*5*_ are only active at *t*_*c*_. This gives a cumulative degree of 5. While this approach captures the breach of an ego’s network over time, it can introduce excessive noise by including transitory or fleeting ties that do not persist, thereby obscuring temporal trends in network dynamics.

To overcome the limitations of the active and cumulative degree measures, we use the method outlined in Miritello et al. (2013) and propose a new method for measuring degree. This approach incorporates both active and persisting ties, providing a more stable representation of an ego’s meaningful connections over time. Specifically, as shown in Fig. 1A (right), in addition to the active alters {*j*_*3*_, *j*_*4*_, *j*_*5*_} at *t*_*c*_ and the cumulative alters {*j*_*1*_, *j*_*2*_, *j*_*4*_} from *t*_*1*_ to *t*_*c*−1_, the ego *i* also has alters {*j*_*2*_, *j*_*4*_, *j*_*6*_} active between the time point after *t*_*c*_ (*t*_*c*+1_) and the last time point (*t*_*l*_).

As shown in Fig. 1B (middle), we first identity the persisting alters, which are those active both before (*t*_*1*_ to *t*_*c*−1_) and after (*t*_*c*+1_ to *t*_*l*_) *t*_*c*_. In this case, the persisting alters are the intersection of {*j*_*1*_, *j*_*2*_, *j*_*4*_} and {*j*_*2*_, *j*_*4*_, *j*_*6*_}, resulting in {*j*_*2*_, *j*_*4*_}. Then, we combine the active alters at *t*_*c*_ {*j*_*3*_, *j*_*4*_, *j*_*5*_} with the persisting alters {*j*_*2*_, *j*_*4*_}. The union of these sets is {*j*_*2*_, *j*_*3*_, *j*_*4*_, *j*_*5*_}. In this set, *j*_*3*_ and *j*_*5*_ are only active at *t*_*c*_, *j*_*4*_ is both an active alter at *t*_*c*_ and a persisting alter before and after *t*_*c*_, and *j*_*2*_ is a persisting alter but not active at *t*_*c*_.

The effective degree for *t*_*c*_ is therefore 4. This method strikes a balance between capturing current connections and accounting for ties that persist over time, reducing noise from transient connections while preserving the temporal granularity necessary for dynamic analysis. As such, the effective degree is particularly well-suited for studies like ours, which examine evolving networks over time.

The calculation of effective degree can be influenced by a boundary effect. Specifically, when the current time point (*t*_*c*_) is too close to *t*_*1*_​ (the first time point) or *t*_*l*_ (the last time point), the intersection of the two sets– one representing interactions from *t*_*1*_​​ to *t*_*c*−1_​, and the other from *t*_*c*+1_ ​ to *t*_*l*_– may contain fewer elements, potentially leading to biased estimates of the effective degree. Conversely, when *t*_*c*_ is farther from both *t*_*1*_​ and *t*_*l*_​, the intersection set may include more elements, potentially influencing temporal trends such as an initial increase followed by a decrease in the mean effective degree.

To address this boundary effect, we carefully selected the time points for our analysis. In this study, *t*_*1*_​ is set to August 23, 2015, the very first day of the Fall 2015 semester, ensuring that the network evolution is captured from its inception. *t*_*c*_ ends on December 19, 2015, the last day of the Fall 2015 semester, avoiding disruptions to temporal patterns caused by the winter break or holiday season. Finally, *t*_*l*_ is set to May 7, 2016, the last day of the 2015–2016 school year, which is well beyond the last *t*_*c*_ (December 19, 2015). This extended time frame allows sufficient time to identify persisting ties, reducing the likelihood of an artificially small intersection set for the second temporal range (*t*_*c*+1_​ to *t*_*l*_​). As a result, the effective degree calculation better reflects meaningful and stable social connections. By strategically defining these time points, we aim to minimize potential biases from varying intersection sizes and ensure more accurate and reliable estimations of the effective degree throughout the observation period.

### Analytical strategy

In this study, the latent growth-curve model (LGCM) is employed to examine the temporal dynamics of the friendship paradox in Stata V18. The LGCM is a statistical modeling technique that allows researchers to investigate how a particular variable changes over time for a group of individuals. It provides a framework for analyzing the within-subject variation of the friendship index in terms of the initial status (intercept), linear growth rate (slope), and quadratic growth patterns, as well as exploring between-subject differences in these patterns.

Mathematically, the Level 1 within-subject equation can be expressed as follows:

2 $$\:{FI}_{it}={\eta\:}_{0i}+{\eta\:}_{1i}\bullet\:t+{\eta\:}_{2i}\bullet\:{t}^{2}+{\beta\:}_{1t}{x}_{it}+{\epsilon\:}_{it}$$

where:

- $\:{FI}_{it}$ represents the observed values of the friendship index for vertex *i* at time $\:t$,
- $\:{\eta\:}_{0i}$ is the intercept, reflecting the initial level of the friendship index at the beginning of the study,
- $\:{\eta\:}_{1i}$ is the linear slope coefficient, representing the average growth rate of the friendship index over time,
- $\:{\eta\:}_{2i}$ is the curvature (quadratic) coefficient, capturing any acceleration or deceleration in the growth trajectory of the friendship index,
- $\:t$ is time (e.g., days in the study),
- $\:{t}^{2}$ is the square of time, allowing the model to capture nonlinear temporal effects,
- $\:{x}_{it}$ is the time-varying variable for vertex *i* at time *t*,
- $\:{\beta\:}_{1t}$ represents the estimated parameter for $\:{x}_{it}$, and
- ∙ $\:{\epsilon\:}_{it}$ represents the residual error term.

The Level 2 between-subject equations can be expressed as follows:

3 $$\:{\eta\:}_{0i}={\gamma\:}_{00}+{\gamma\:}_{01}{z}_{i}+{\delta\:}_{0i}$$

4 $$\:{\eta\:}_{1i}={\gamma\:}_{10}+{\gamma\:}_{11}{z}_{i}+{\delta\:}_{1i}$$

5 $$\:{\eta\:}_{2i}={\gamma\:}_{20}+{\gamma\:}_{21}{z}_{i}+{\delta\:}_{2i}$$

where:

- $\:{\gamma\:}_{*0}$ denotes the intercept terms,
- $\:{z}_{i}$ is the time-constant variable (e.g., gender, race/ethnicity, the Big Five personality factors, and other psychological factors),
- $\:{\gamma\:}_{*1}$ are coefficients representing the effects of the time-constant variable $\:{z}_{i}$ on the intercept and the linear and quadratic growth rates, respectively, and
- $\:{\delta\:}_{*i}$ accounts for between-subject random error terms.

In the context of the friendship paradox, the LGCM is used to examine how the friendship index, representing the degree disparity between egos and their alters, changed over the course of the semester. By estimating the intercept, linear, and quadratic growth coefficients, the model provided insights into the initial level, rate of change, and curvature of the friendship index. Additionally, the LGCM enables the exploration of potential factors influencing the friendship paradox by including both time-constant and time-varying predictors in the model. Through this statistical approach, the study aimed to uncover the trajectory of the friendship paradox and investigate the role of various factors in its manifestation and development over time.

## Results

### Descriptive statistics

**Fig. 2:**
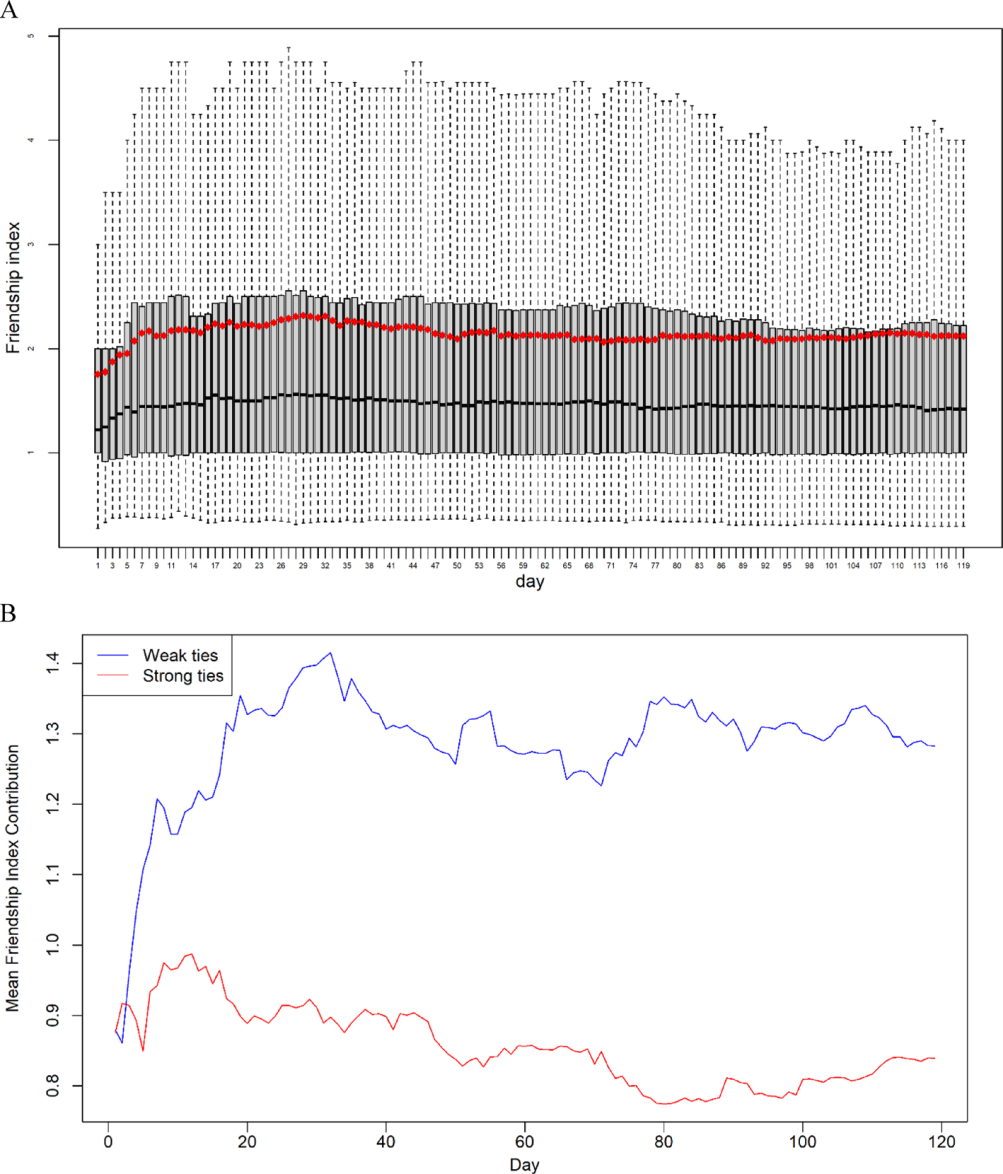
Temporal trend of the friendship index from 08/23/2015 to 12/19/2015, showing its stabilization after an initial increase and the greater contribution of weak ties to the friendship index compared to strong ties

Figure 2A shows the temporal pattern of the friendship index over the course of 119 days. Each day is represented by a box, displaying the 25th-75th percentiles. Within the box, the dark line represents the median, or the 50th percentile. The dashed lines above and below the box depict the 10th-90th percentiles. Additionally, the red diamond represents the mean value of the friendship index. Analyzing the graph, we observe that on the initial day, the median of the friendship index is approximately 1.2. It gradually increases to around 1.4 by the 7th day and then remains relatively stable. As for the mean friendship index, it starts at approximately 1.8 on the first day and surpasses 2 by the 7th day. Although some fluctuations occur afterward, the overall trend is relatively stable. Consequently, we observe that the friendship paradox emerges from the very beginning, and it takes approximately one week for the friendship index to reach a stable state.

Figure 2B illustrates the contribution of ties with varying strengths to the friendship index. We classify 25% of the 9,732 phone interaction ties that were also reported in the Qualtrics surveys as strong ties, while the remaining 75% are categorized as weak ties. The contribution of strong ties, represented in red, is calculated as $\:{FI}_{{i}_{strong}}=\frac{{\sum\:}_{j\in\:{V}_{strong}}{d}_{j}}{{d}_{i}^{2}}$, while the contribution of weak ties, shown in blue, is computed as $\:{FI}_{{i}_{weak}}=\frac{{\sum\:}_{j\in\:{V}_{weak}}{d}_{j}}{{d}_{i}^{2}}$. Figure 2B shows the mean $\:{FI}_{{i}_{strong}}$ and $\:{FI}_{{i}_{weak}}$ values across the 119 days, highlighting that weak ties contribute more significantly to the friendship index than strong ties, a finding further confirmed by a paired *t*-test. It is important to note that the denominator for both $\:{FI}_{{i}_{strong}}$ and $\:{FI}_{{i}_{weak}}$ is $\:{d}_{i}^{2}$. While the mean $\:{FI}_{{i}_{weak}}$ generally exceed 1 and the mean $\:{FI}_{{i}_{strong}}$ values remain below 1, this does not necessarily imply that strong-tie alters have fewer friends than the ego. Rather, it suggests that the sum of their degrees (i.e., their total connectivity in the network) is disproportionately smaller relative to the ego’s squared degree, resulting in a lower contribution to the overall friendship index. Conversely, weak ties, due to their higher variability and number, have a stronger amplifying effect on the friendship index.

**Fig. 3:**
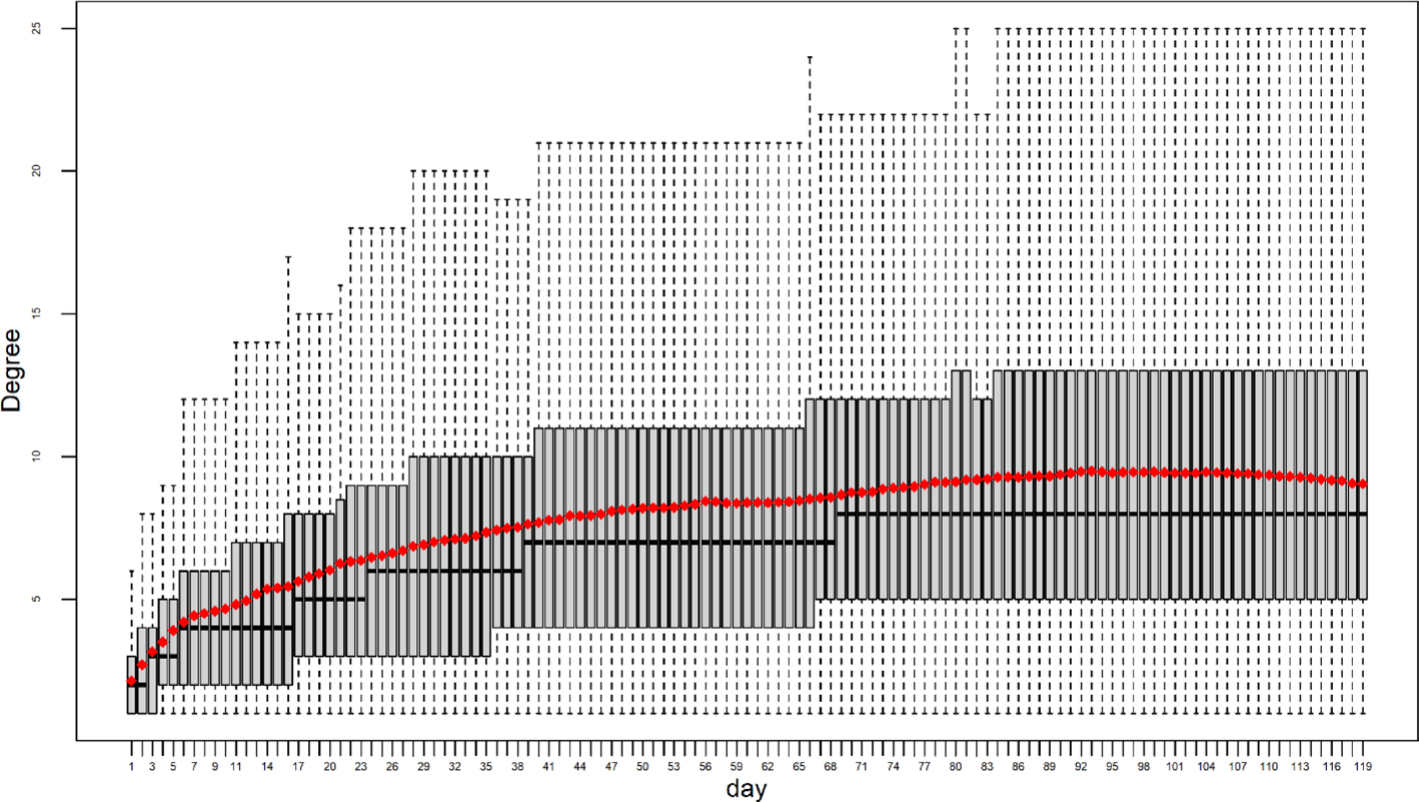
Temporal trend of effective degree from 08/23/2015 to 12/19/2015, demonstrating its continuous growth throughout the study period

The equation of the friendship index suggests a non-linear relationship with the effective degree, possibly quadratic in nature. Therefore, it is also worth examining the temporal pattern of the effective degree. The box plot in Fig. 3 presented here demonstrates that the median degree of an ego shows a consistent upward trend: it starts at 2 on day 1 and day 2, increases to 3 from day 3 to day 5, further rises to 4 from day 6 to day 16, reaches 5 from day 17 to day 23, grows to 6 from day 24 to day 38, expands to 7 from day 39 to day 68, and finally reaches 8 from day 69 till day 119. Similarly, the mean degree follows a similar pattern: a steep increase at the beginning, followed by a continued rise, albeit at a slower rate over time.

**Fig. 4:**
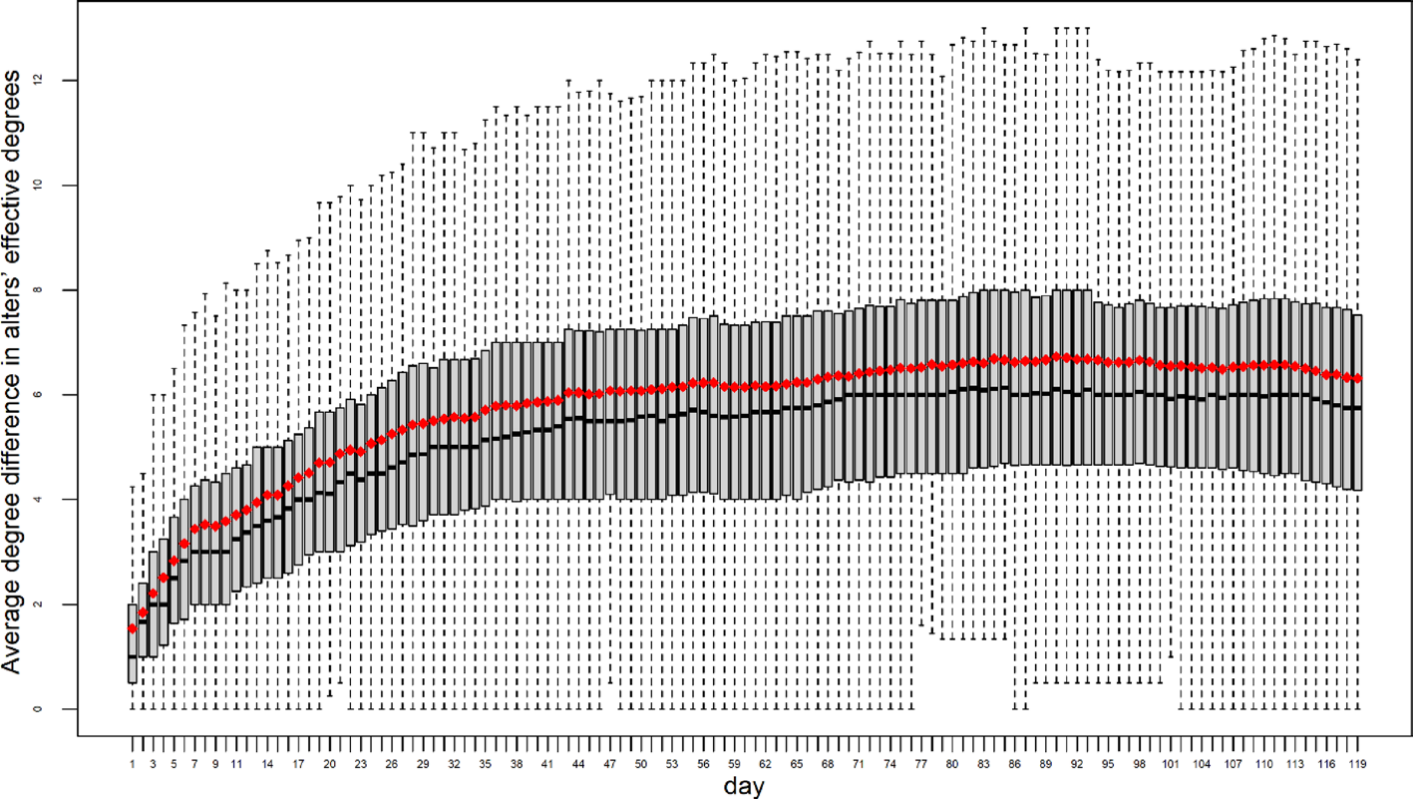
Changes in the average degree difference in alters’ effective degrees from 08/23/2015 to 12/19/2015, highlighting increasing degree heterogeneity over time

Another important question to consider is whether the effective degrees of alters are heterogeneous. The friendship index captures the degree disparity between an ego and their alters, and this disparity can be influenced by variations in the alters’ degrees. If some alters are highly connected while others have low connectivity, this variation can amplify the degree disparity, thereby affecting the friendship index. To investigate this, we examine the average degree difference, or standard deviation, across an ego’s alters. As shown in Fig. 4, the average degree difference increases sharply at the beginning and continues to rise, although the rate of increase slows over the 119-day period. This pattern suggests that, on average, egos in the smartphone communication network have alters with both low and high degrees, contributing to the observed degree heterogeneity.

**Fig. 5:**
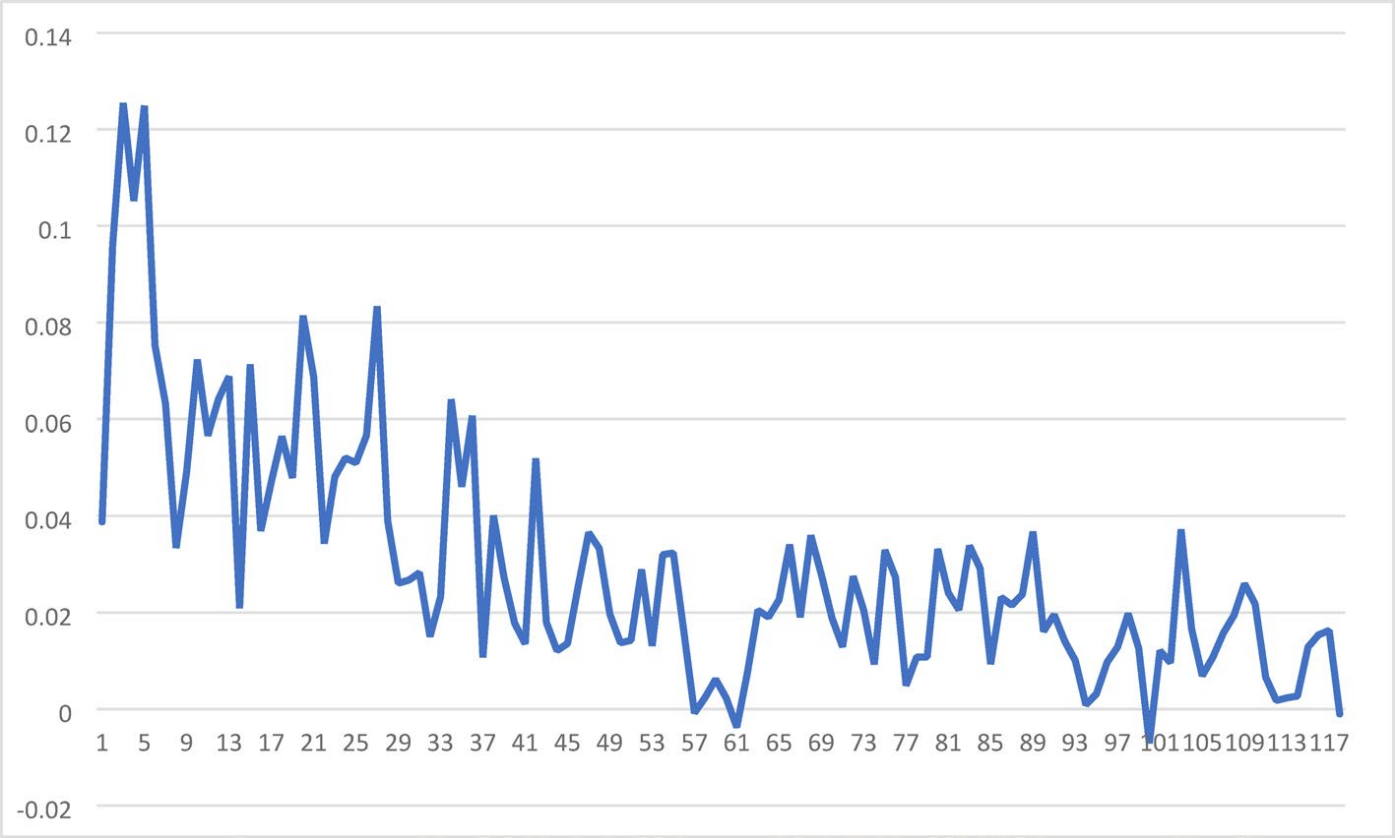
Log-log relationship between degree and newly formed ties, illustrating the extent to which individuals with higher degrees continue to form new connections

To assess the presence of preferential attachment in the smartphone communication network, we calculated the number of new ties ($\:{d}_{it}^{new}$​) formed by an ego with a given effective degree ($\:{d}_{it}$​) on a daily basis during the study period. We then performed a series of linear regression of $\:{\text{l}\text{o}\text{g}(d}_{it}^{new})$ on $\:\text{l}\text{o}\text{g}\left({d}_{it}\right)$ each day. Figure 5 shows the daily log-log coefficients between degree and new ties, which are positive on most days, with only four exceptions. This consistent positive relationship indicates that individuals with higher degrees are more likely to form new connections, providing strong evidence of preferential attachment. The strength of this log-log relationship varies over time, with higher coefficients observed in the early days of the study, suggesting that preferential attachment was more pronounced during the initial phase of network formation. Over time, the coefficients decline, indicating that the influence of preferential attachment weakens as the network matures, eventually plateauing at lower but still positive values. This persistent, albeit reduced, influence highlights the enduring role of preferential attachment in the network.

### Friendship metrics by demographic, personality, and psychological factors

Figures 6 A to 17 C, located in the Appendix, consistently demonstrate that the friendship index, effective degree, and average degree difference in alters’ effective degrees increased over the 119 days, though their growth rates gradually slowed over time. These trends are examined across gender, race/ethnicity, religious preference, Big Five personality traits, and other psychological characteristics, including self-esteem, loneliness, self-regulation, and depression.

Figures 6 A to 6 C (in the Appendix) illustrate gender-based differences. On the first day, females exhibited a higher friendship index and slightly lower effective degree. However, from the second day onward, females maintained a lower friendship index but a higher effective degree compared to males. Additionally, starting from day 21, females displayed a higher average degree difference in alters’ effective degrees than their male counterparts.

Figures 7 A to 7 C (in the Appendix) highlight patterns across race and ethnicity. Latino and Black students had higher friendship indices on the first day, while Asian and foreign students had lower values compared to White students. After the first day, foreign students showed an increase in their friendship index, surpassing other groups, while Black students had a lower friendship index, with White, Latino, and Asian students positioned between these extremes. The effective degree started similarly across all groups, but Black students eventually matched White students, followed by Latino, Asian, and foreign students. The average degree difference in alters’ effective degrees was generally comparable across White, Latino, Black, and Asian students, while it remained lower for foreign students.

Figures 8 A to 8 C (in the Appendix) depict trends based on religious preference. Catholic and Protestant students began with higher friendship indices than students identifying with no religion or other religions. However, over time, Protestant students, along with those from other and no religious affiliations, surpassed Catholic students in friendship index. In terms of effective degree, Catholic students maintained the highest values, followed by Protestant students and those with no religion, while students of other religions consistently exhibited the lowest effective degree. The average degree difference in alters’ effective degrees was consistent across all religious groups throughout the observation period.

For each of the Big Five personality traits and other psychological factors, students are categorized into low, moderate, and high groups, with each group containing an equal number of students. Figures 9 A to 9 C (in the Appendix) reveal differences in the Big Five personality trait of extraversion. Students with low extraversion exhibited a higher friendship index, lower effective degree, and higher average degree difference in alters’ effective degrees. Conversely, students with high extraversion showed lower friendship indices, higher effective degrees, and moderate average degree differences. Those with moderate extraversion demonstrated a moderate friendship index, moderate effective degree, and lower average degree differences in alters’ effective degrees.

Figures 10 A to 10 C (in the Appendix) indicate that students with low agreeableness had a higher friendship index, lower effective degree, and higher average degree difference in alters’ effective degrees. Those with moderate agreeableness displayed a lower friendship index, moderate effective degree, and lower average degree differences, while students with high agreeableness exhibited a moderate friendship index, higher effective degree, and moderate average degree difference.

No consistent trends emerged across different levels of conscientiousness, as shown in Figs. 11 A to 12 C (in the Appendix), for the friendship index, effective degree, or average degree difference in alters’ effective degrees. However, Figs. 12 A to 12 C (in the Appendix) indicate that students with high neuroticism displayed a higher friendship index, lower effective degree, and higher average degree difference compared to students with lower or moderate neuroticism. Figures 13 A to 13 C (in the Appendix) suggest that students with moderate openness had a friendship index comparable to those with low and high openness but exhibited a lower effective degree and average degree difference in alters’ effective degrees.

Other psychological factors also revealed interesting patterns. Figures 14 A to 14 C (in the Appendix) show that students with high self-esteem had a higher friendship index, lower effective degree, and higher average degree difference in alters’ effective degrees compared to students with low or moderate self-esteem. Figures 15 A to 15 C (in the Appendix) highlight that students with low loneliness demonstrated a higher friendship index, lower effective degree, and higher average degree difference than those with moderate or high loneliness. However, no consistent patterns were observed for self-regulation across any of the metrics, as illustrated in Figs. 16 A to 16 C (in the Appendix). Finally, Figs. 17 A to 17 C (in the Appendix) reveal that students with high depression exhibited a higher friendship index, lower effective degree, and higher average degree difference compared to those with low or moderate depression.

In summary, the findings reveal significant variations in friendship index, effective degree, and average degree difference in alters’ effective degrees across gender, race/ethnicity, religious preferences, personality traits, and psychological characteristics. These trends underscore the complexity of the friendship paradox and its relationship with individual traits and social behaviors in a smartphone-based communication network.

### LGCM results

Table 3 displays the results of the latent growth-curve model (LGCM). The model exhibits a good fit, as indicated by the Root Mean Square Error of Approximation (RMSEA) value below 0.06 and the Comparative Fit Index (CFI) value exceeding 0.95.

**Table 3:** Results from the latent growth-curve model examining the Temporal dynamics of the friendship index, including estimates for initial levels, linear growth, and quadratic growth patterns

	Model 1		Model 2	
	Parameter	95% confidence interval	Parameter	95% confidence interval
Day	0.058***	(0.055, 0.061)	0.057***	(0.054, 0.060)
Day^2^	-0.000***	(-0.000, -0.000)	-0.000***	(-0.000, -0.000)
$\:{d}_{i}$	-0.540***	(-0.549, -0.530)	-0.560***	(-0.571, -0.550)
$\:{d}_{i}^{2}$	0.015***	(0.015,0.016)	0.016***	(0.016,0.016)
Proportion of weak ties			0.535***	(0.463, 0.607)
Female (1 = yes)	3.085***	(2.642, 3.529)	2.928***	(2.498, 3.358)
Female × Day	-0.020***	(-0.026, -0.015)	-0.019***	(-0.025, -0.014)
Female × Day^2^	-0.000***	(-0.000, -0.000)	-0.000***	(-0.000, -0.000)
Latino (1 = yes)	3.148***	(2.401, 3.896)	3.026***	(2.302, 3.751)
Latino × Day	-0.032***	(-0.040, -0.024)	-0.031***	(-0.039, -0.023)
Latino × Day^2^	0.000***	(0.000, 0.000)	0.000***	(0.000, 0.000)
African American (1 = yes)	0.705	(-0.487, 1.899)	0.641	(-0.515, 1.797)
African American × Day	-0.012	(-0.025, 0.000)	-0.012	(-0.024, 0.001)
African American × Day^2^	-0.000	(-0.000, 0.000)	-0.000	(-0.000, 0.000)
Asians American (1 = yes)	1.517**	(0.608, 2.427)	1.409**	(0.527, 2.290)
Asians American × Day	-0.016**	(-0.027, -0.007)	-0.016**	(-0.026, -0.007)
Asians American × Day^2^	0.000	(-0.000, 0.000)	0.000	(-0.000, 0.000)
Foreign student (1 = yes)	0.479	(-0.575, 1.534)	0.443	(-0.578, 1.465)
Foreign student × Day	-0.002	(-0.014, 0.009)	-0.003	(-0.014, 0.008)
Foreign student × Day^2^	-0.000***	(-0.000, -0.000)	-0.000***	(-0.000, -0.000)
Protestant (1 = yes)	1.438**	(0.585, 2.292)	1.349***	(0.522, 2.176)
Protestant × Day	0.007	(-0.001, 0.017)	0.008	(-0.001, 0.017)
Protestant × Day^2^	-0.000***	(-0.000, -0.000)	-0.000***	(-0.000, -0.000)
Other religion (1 = yes)	1.900**	(0.576, 3.224)	1.779**	(0.496, 3.061)
Other religion × Day	-0.029***	(-0.043, -0.015)	-0.029***	(-0.043, -0.015)
Other religion × Day^2^	0.000***	(0.000, 0.000)	0.000***	(0.000, 0.000)
No religion (1 = yes)	2.019***	(1.213, 2.826)	1.921***	(1.139, 2.702)
No religion × Day	-0.011**	(-0.020, -0.003)	-0.011**	(-0.019, -0.002)
No religion × Day^2^	0.000*	(0.000, 0.000)	0.000*	(0.000, 0.000)
Extraversion	-0.124	(-0.519, 0.272)	-0.138	(-0.522, 0.245)
Extraversion × Day	-0.002	(-0.006, 0.002)	-0.002	(-0.006, 0.002)
Extraversion × Day^2^	0.000***	(0.000, 0.000)	0.000***	(0.000, 0.000)
Agreeableness	-0.868***	(-1.327, -0.409)	-0.830***	(-1.274, -0.385)
Agreeableness × Day	0.007**	(0.002, 0.012)	0.007**	(0.002, 0.012)
Agreeableness × Day^2^	0.000	(-0.000, 0.000)	0.000	(-0.000, 0.000)
Conscientiousness	-0.495	(-1.043, 0.053)	-0.500	(-1.031, 0.031)
Conscientiousness × Day	0.004	(-0.002, 0.010)	0.004	(-0.002, 0.010)
Conscientiousness × Day^2^	0.000**	(0.000, 0.000)	0.000**	(0.000, 0.000)
Neuroticism	-0.745**	(-1.265, -0.225)	-0.702**	(-1.206, -0.198)
Neuroticism × Day	0.011***	(0.005, 0.017)	0.011***	(0.005, 0.016)
Neuroticism × Day^2^	-0.000***	(-0.000, -0.000)	-0.000***	(-0.000, -0.000)
Openness	-0.050	(-0.507, 0.407)	-0.045	(-0.487, 0.398)
Openness × Day	-0.007**	(-0.012, -0.002)	-0.007**	(-0.011, -0.002)
Openness × Day^2^	0.000***	(0.000, 0.000)	0.000***	(0.000, 0.000)
Self-esteem	-0.387	(-0.896, 0.123)	-0.361	(-0.855, 0.132)
Self-esteem × Day	-0.006*	(-0.011, -0.000)	-0.006*	(-0.011, -0.000)
Self-esteem × Day^2^	0.000***	(0.000, 0.000)	0.000***	(0.000, 0.000)
Loneliness	-0.495*	(-0.988, -0.002)	-0.502*	(-0.979, -0.024)
Loneliness × Day	0.013***	(0.008, 0.018)	0.013***	(0.008, 0.018)
Loneliness × Day^2^	-0.000***	(-0.000, -0.000)	-0.000***	(-0.000, -0.000)
Self-regulation	-0.488	(-1.090, 0.114)	-0.495	(-1.078, 0.088)
Self-regulation × Day	0.000	(-0.006, 0.006)	0.001	(-0.006, 0.007)
Self-regulation × Day^2^	0.000**	(0.000, 0.000)	0.000*	(0.000, 0.000)
Depression	0.529	(-0.089, 1.148)	0.501	(-0.098, 1.100)
Depression × Day	0.003	(-0.004, 0.009)	0.003	(-0.004, 0.009)
Depression × Day^2^	-0.000	(-0.000, 0.000)	-0.000	(-0.000, 0.000)
Saturday	0.033***	(0.015,0.051)	0.034***	(0.016,0.052)
Sunday	-0.008	(-0.026, 0.011)	-0.007	(-0.025, 0.011)
Midterm break	-0.129***	(-0.155, -0.104)	-0.128***	(-0.153, -0.103)
Thanksgiving holiday	-0.012	(-0.044, 0.020)	-0.014	(-0.046, 0.018)
Number of cases	77,433		77,433	
Number of individuals	669		669	
Goodness-of-fit				
AIC	209829.5		209622.8	
BIC	210412.7		210215.3	
Wald chi-square/df	14527.06/59		14801.31/60	
Log-likelihood	-104851.73		-104747.42	

Note: (1) *p < 0.05, **p < 0.01, ***p < 0.001; (2) The constant term is suppressed to zero in the model

In Model 1, the intercept [$\:{\eta\:}_{0i}$ in Eq. (2)], representing the initial level of friendship index on the first day, is assumed to be 0. Our analysis reveals that the friendship index demonstrates an overall increasing trend during this period. The positive linear effect [$\:{\eta\:}_{1i}$ in Eq. (2)] indicates a gradual growth in the friendship index as time progresses. Moreover, the negative quadratic effect [$\:{\eta\:}_{2i}$ in Eq. (2)] highlights a declining growth rate of the friendship index over time, indicating that while the friendship index continues to rise, the rate of growth gradually diminishes as the study period unfolds.

Nodal degree is a time-varying variable [$\:{x}_{it}$ in Eq. (2)] due to its daily fluctuations. Examining the effect of degree [$\:{\beta\:}_{1t}$ in Eq. (2)] on the friendship index, our analysis indicates that a higher degree is associated with a lower friendship index. This finding suggests that individuals with a larger number of connections tend to have a reduced likelihood of experiencing the friendship paradox. However, it is noteworthy that the negative effect of degree on the friendship index is not linear. The negative squared effect of degree ($\:{\beta\:}_{1t}$) indicates that as the degree increases further, the negative impact on the friendship index diminishes. In other words, individuals with exceptionally high degrees may experience a weakening of the negative relationship between degree and the manifestation of the friendship paradox. These results provide empirical support for both the linear and nonlinear effects of degree on the friendship index, as proposed in Eq. (1).

Personal demographic factors, including gender, race/ethnicity, and religious preference, are time-constant variables [$\:{z}_{i}$ in Eq. (3) to (5)]. In Stata V18, the intercept [$\:{\gamma\:}_{00}$ in Eq. (3)], linear growth rate [$\:{\gamma\:}_{10}$ in Eq. (4)], and quadratic growth rate [$\:{\gamma\:}_{20}$ in Eq. (5)] of the reference group or baseline group are set to be 0. Our analysis reveals that females have a higher initial level of the friendship index [$\:{\gamma\:}_{01}$ in Eq. (3)] compared to males, but this index decreases over time [$\:{\gamma\:}_{11}$ in Eq. (4)]. Similar patterns emerge when considering ethnic backgrounds. Latinos and Asian Americans start with higher friendship indices ($\:{\gamma\:}_{01}$) compared to White participants, but their indices tend to decrease over time ($\:{\gamma\:}_{11}$). Moreover, comparing religious preferences, participants who identify with religions other than Catholicism exhibit higher initial levels of the friendship index ($\:{\gamma\:}_{01}$). However, a divergent temporal pattern emerges, with participants of other religious affiliations and those with no religious preferences experiencing a decreasing trend over time ($\:{\gamma\:}_{11}$).

The Big Five personality factors and other psychological factors are also time-constant variables [$\:{z}_{i}$ in Eq. (3) to (5)]. Our analysis reveals that individuals with higher levels of agreeableness, neuroticism, and loneliness tend to have a lower initial level of the friendship index ($\:{\gamma\:}_{01}$), but they experience a positive growth rate over time ($\:{\gamma\:}_{11}$). On the other hand, self-esteem does not show a significant association with the initial state of the friendship index ($\:{\gamma\:}_{01}$) but is negatively related to its growth rate ($\:{\gamma\:}_{11}$). It should be noted that the effects of all time-constant variables on quadratic growth rates [$\:{\gamma\:}_{21}$ in Eq. (5)] are minimal, indicating limited influence on curvilinear growth patterns.

The analysis of other time-varying variables [$\:{x}_{it}$ in Eq. (2)] reveals interesting findings regarding the friendship index. We observe that the friendship index tends to increase on Saturdays ($\:{\beta\:}_{1t}$) compared to weekdays, suggesting a higher likelihood of encountering the friendship paradox during weekends. Additionally, we find a decrease in the friendship index during midterm breaks ($\:{\beta\:}_{1t}$) compared to regular school days. These findings highlight how temporal factors, such as academic schedules and weekends, shape the manifestation of the friendship paradox.

In Model 2, we incorporate the proportion of weak ties (i.e., phone interaction ties that were ***not*** reported in the Qualtrics surveys) per day as a time-varying variable [$\:{x}_{it}$ in Eq. (2)]. Our analysis reveals a positive effect of weak ties on the friendship index, suggesting that their greater variability in number, compared to strong ties, contributes to degree disparity and amplifies the friendship paradox. Importantly, the significance patterns of all other variables remain consistent with those observed in Model 1.

## Discussion

The findings from this analysis reveal fascinating patterns regarding the friendship paradox in the smartphone communication network. The initial observation of the friendship index consistently exhibiting values above 1 from the very first day indicates that an ego’s friends tend to have more friends on average. This observation aligns with the notion of the friendship paradox, where individuals in a network are likely to have fewer friends on average compared to their friends. This finding is in line with the 80 − 20 rule (Pareto 1964), which suggests that a small fraction of individuals in a network tend to have a disproportionately large number of connections.

As the days progressed, the values of the friendship index continued to increase over the first week, indicating a growing disparity in degree between the ego and their alters. This suggests that the ego kept connecting to well-connected individuals and these highly connected alters were gaining even more connections, amplifying the friendship paradox phenomenon within the network. This growth pattern is consistent with the preferential attachment theory (Barabási and Albert 1999), which posits that new connections in a network are more likely to be formed with individuals who already have a high degree, thus reinforcing the existing hierarchy of connections.

After this initial growth phase, the friendship index achieved stability and remained relatively constant for the rest of the semester. However, it is noteworthy that while the friendship index stabilized, both the ego’s own degree and the variation among their friends’ degrees continued to increase throughout the semester. This indicates that the ego and their alters actively formed new connections and expanded their social circles over time, highlighting the dynamic nature of the network and individuals’ efforts to forge new relationships.

Here, the most striking observation is that the friendship index reached its equilibrium state much faster than the measures of degree. This suggests underlying mechanisms driving this rapid stabilization. One plausible explanation is preferential attachment dynamics within the network (Barabási and Albert 1999). During the early stages of network formation, egos forming new connections tend to link with well-connected individuals, resulting in a rapid initial rise in the friendship index. This aligns with our findings of a consistently positive relationship between an individual’s degree and their likelihood of forming new ties, providing strong evidence of preferential attachment. However, as the network matures, the influence of preferential attachment diminishes, reducing the impact of connections to highly connected alters on the friendship index. This waning effect explains why the friendship index converges more quickly than the ego’s degree or the variation in their friends’ degrees. Preferential attachment drives the early acceleration of the friendship index, but its reduced influence facilitates the faster stabilization, highlighting its critical role in the temporal dynamics and evolution of the network.

Another explanation could relate to small-world theory, which suggests that networks often display a high degree of interconnectedness, allowing most individuals to be reached through just a few intermediate connections (Milgram 1967; de Sola Pool and Kochen 1978; Watts and Strogatz 1998). This efficient structure may limit the further expansion of the friendship paradox. As the network grows and more connections are established, it eventually reaches a saturation point where most individuals are accessible through a small number of intermediaries. At this stage, the potential for individuals to have friends with exceptionally high degrees becomes constrained, as the network’s efficiency ensures that most people are already well-connected through their immediate contacts. This interconnectedness reduces the likelihood of having alters with significantly higher degrees, thereby curtailing the extent to which the friendship paradox can intensify. Consequently, the network’s intrinsic structure and efficiency help to stabilize the degree disparity and limit the further expansion of the friendship paradox.

Our latent growth-curve model reveals other notable findings, including the dual relationship between degree and the friendship index– specifically, a negative linear relationship alongside a positive quadratic relationship. This finding is consistent with the definition in Eq. (1), where, as the denominator (the ego’s degree) increases, the friendship index decreases. However, the rate of this decrease slows as the degree continues to grow. This dual relationship underscores the nuanced and evolving nature of social networks, illustrating how degree disparities and the types of connections individuals form influence the manifestation of the friendship paradox.

The examination of personal demographic factors revealed compelling patterns as well. The gender difference in the friendship index suggests that females exhibit a distinct communication pattern compared to males. Initially, females have more connections with well-connected individuals, resulting in a higher starting level of the friendship index. However, over time, females are less likely to maintain this pattern, leading to a lower growth rate of the friendship index compared to males. These gender-specific patterns reflect differences in communication styles and social dynamics between males and females as in existing literature (Moore 1990; Walker 1994; Igarashi et al. 2005).

Ethnic backgrounds also played a significant role in shaping the friendship index. Latinos and Asian Americans began the semester with higher friendship indices compared to White participants, which may be attributed to cultural preferences for larger social circles or the influence of close-knit community networks (Kwon et al. 2013; Barajas 2020). This pattern aligns with the idea about how racial and ethnic minorities balance networks that include both in-group connections (with other minorities) and out-group connections (with majority members), resulting in diverse and heterogeneous social interactions that could amplify the friendship paradox (Blau 1977). However, their slower growth rates in the friendship index suggests a focus on stability, loyalty, and long-term relationships, consistent with cultural norms that prioritize enduring connections over frequent social expansion (Yamamoto and Acosta 1982; Marín 1993; Suizzo 2007). This finding highlights how cultural values shape the pace and nature of network evolution, with minority groups initially leveraging existing in-group ties before gradually integrating into broader networks. Understanding these dynamics has broader implications for fostering social cohesion and inclusivity in diverse environments, as it underscores the need to consider cultural influences when interpreting network structures and their development.

Similarly, the divergent temporal patterns observed among individuals with different religious preferences emphasize the impact of cultural norms and community structures on friendship formation and maintenance. The influence of religious affiliation on the friendship index suggests that individuals with different religious backgrounds may have distinct patterns of social connections and networks, influenced by their respective religious communities.

Regarding psychological factors, individuals with higher levels of agreeableness, neuroticism, and loneliness demonstrated a lower initial level of the friendship index. This suggests that these individuals may have smaller social circles or be more selective in forming close friendships. Possible explanations for this pattern could be that highly agreeable individuals may prioritize quality over quantity in their social connections, seeking meaningful and deep relationships rather than a large number of superficial connections (Asendorpf and Wilpers 1998; Knack et al. 2013). Similarly, individuals with higher levels of neuroticism may be more cautious in forming new friendships, potentially due to fear of rejection or concerns about maintaining emotional well-being in interpersonal relationships (Asendorpf and Wilpers 1998; Premkumar et al. 2015). Furthermore, individuals experiencing higher levels of loneliness may have difficulty forming and maintaining social connections, resulting in a lower initial friendship index (Heinrich and Gullone 2006; Musetti et al. 2019).

However, individuals with higher levels of agreeableness, neuroticism, and loneliness also exhibited a positive growth rate for the friendship index. This suggests that over time, these individuals actively seek out new friendships and expand their social circles. It is possible that individuals with higher levels of agreeableness actively engage in prosocial behaviors and display friendliness, making it easier for them to form new connections as they become more comfortable with their social environment (Asendorpf and Wilpers 1998; Knack et al. 2013). Similarly, individuals with higher levels of neuroticism may gradually overcome their initial shyness and develop new friendships as they become more familiar and comfortable in social situations (Asendorpf and Wilpers 1998; Premkumar et al. 2015). Individuals experiencing higher levels of loneliness may actively seek social interactions and work towards reducing their feelings of isolation (Heinrich and Gullone 2006; Musetti et al. 2019), leading to an increase in the friendship index over time.

In contrast, individuals with higher levels of self-esteem demonstrated a negative growth rate of the friendship index. This suggests a preference for maintaining a stable network or a decrease in the number of connections over time. One possible explanation is that individuals with higher self-esteem may be more selective in choosing their friends, preferring quality over quantity. As their self-esteem increases, they may prioritize maintaining deep and meaningful relationships, leading to a decrease in the number of connections but an increase in the overall quality and satisfaction of their social interactions (Marshall et al. 2014; Harris and Orth 2020).

The analysis of time-varying variables provides further insights into the friendship paradox. The increase in the friendship index on Saturdays compared to weekdays supports the idea that individuals may have more leisure time and opportunities for social interactions on weekends, leading to a higher likelihood of encountering the friendship paradox. Conversely, the decrease in the friendship index during midterm breaks suggests that the temporary interruption in regular academic activities may lead to reduced face-to-face interactions and individuals distancing themselves from their social networks. This finding reflects the influence of temporal factors, such as academic schedules and breaks, on the dynamics of the friendship paradox.

In addition to these findings, our analysis underscores the significant role of weak ties in shaping the friendship index. Weak ties, which are more numerous and variable than strong ties, contribute more to degree disparity and, consequently, to the amplification of the friendship paradox. Because weak ties often connect individuals with a wider range of degrees, they introduce greater heterogeneity into the network, increasing the likelihood that an individual’s friends appear more socially connected than they are. This suggests that the paradox is not only a function of degree disparity but also of the structural composition of one’s network. Individuals with a higher proportion of weak ties may experience a stronger manifestation of the friendship paradox, as their network connections are more diverse and less constrained by the homophily typically found in strong ties. These findings highlight the importance of differentiating between tie strength when examining social network dynamics and provide further insight into the mechanisms that sustain and amplify the friendship paradox in real-world communication networks.

### Limitations and future research

This study is subject to a couple of limitations that warrant acknowledgement. Firstly, the analysis was conducted using data collected from a single university campus, which may limit the generalizability of the findings to other networks. Replicating the study across diverse contexts, such as online social networks, workplace networks, or community networks, would enhance the external validity of the results and provide a more comprehensive understanding of the friendship paradox in varying social settings. Secondly, while the communication data collected via smartphones occurred in real time, the reliance on self-reported subjective assessments of psychological traits introduces the possibility of recall biases. Future research should seek to incorporate more objective and accurate measures of individuals’ psychological characteristics to enhance the reliability and validity of the findings. Addressing these limitations and conducting future research that encompasses diverse and complete networks and incorporates objective measures in data collection will advance our understanding of the friendship paradox and its implications for social network dynamics.

At the same time, analyzing the mesoscopic and macroscopic structures of the network could offer deeper insights into the dynamics of the friendship paradox. While this study focused on two-step neighborhoods to assess individual-level patterns– such as the roles of nodal degree, variation in alters’ degrees, and preferential attachment in the evolving friendship index– future research could explore mesoscopic features like modularity, clustering, and network motifs (e.g., triangles and bridges). On a macroscopic scale, examining metrics such as degree distributions, assortativity, and centralization could uncover how broader network cohesion, fragmentation, or topology influence the friendship paradox.

These directions represent promising avenues for future work. As noted earlier, the evolving nature of the friendship paradox remains underexplored, in part due to the limited availability of suitable temporal network data. We hope that future datasets, similar to the NetHealth data, will enable systematic investigations of how mesoscopic and macroscopic structures shape the dynamics of the friendship paradox. Expanding research in this area will deepen our understanding of social network dynamics and the broader implications of this intriguing phenomenon.

## Conclusions

In conclusion, this study has significantly deepened our understanding of the temporal dynamics and factors that shape the friendship index, offering new insights into the manifestation and development of the friendship paradox within social networks. The consistent patterns observed– where individuals tend to have fewer friends on average than their friends– highlight the complex nature of social interactions. By analyzing factors such as network growth, individual degrees, personal demographics, psychological traits, and temporal variables, this study has uncovered the intricate interplay that influences the friendship index over time. The integration of key theories, including the 80 − 20 rule, preferential attachment theory, small-world theory, and degree disassortativity theory, has provided valuable explanations for these patterns and enriched our understanding of social network dynamics. These findings carry important implications for disciplines such as sociology, psychology, and network science. In sociology, the study enhances our understanding of the structural dynamics within networks, particularly the mechanisms that amplify the friendship paradox and distort perceptions of social norms and behaviors. In psychology, the findings highlight the role of individual traits– such as agreeableness, neuroticism, loneliness, and self-esteem– in shaping social connections and influencing how individuals perceive their relationships. Lastly, in network science, this research advances our comprehension of network growth processes and the evolving dynamics of social relationships, demonstrating how degree disparities can lead to the disproportionate influence of highly connected individuals on group behavior and perceptions. Overall, this study provides a foundation for exploring the temporal dynamics of the friendship paradox, opening new avenues for future research to investigate the underlying mechanisms and explore additional factors that influence friendship formation and maintenance. Additionally, our findings highlight the critical role of weak ties in amplifying the friendship paradox, demonstrating that individuals with a higher proportion of weak ties are more susceptible to experiencing greater degree disparity in their networks.

## Electronic supplementary material

Below is the link to the electronic supplementary material.

Supplementary Material 1

## Data Availability

The NetHealth data are publicly accessible via the following URL: http://sites.nd.edu/nethealth/data-2/.
