# Supplementary material for: Temporal dynamics of the friendship paradox in a smartphone communication network
Source: Appl Netw Sci. 2025 May 23;10(1):16. doi: 10.1007/s41109-025-00710-1 (PMC12102006; doi:10.1007/s41109-025-00710-1)
Supplement: Supplementary file 1 — Supplementary Material 1 [file 41109_2025_710_MOESM1_ESM.docx]

Appendix A: Additional plots

A


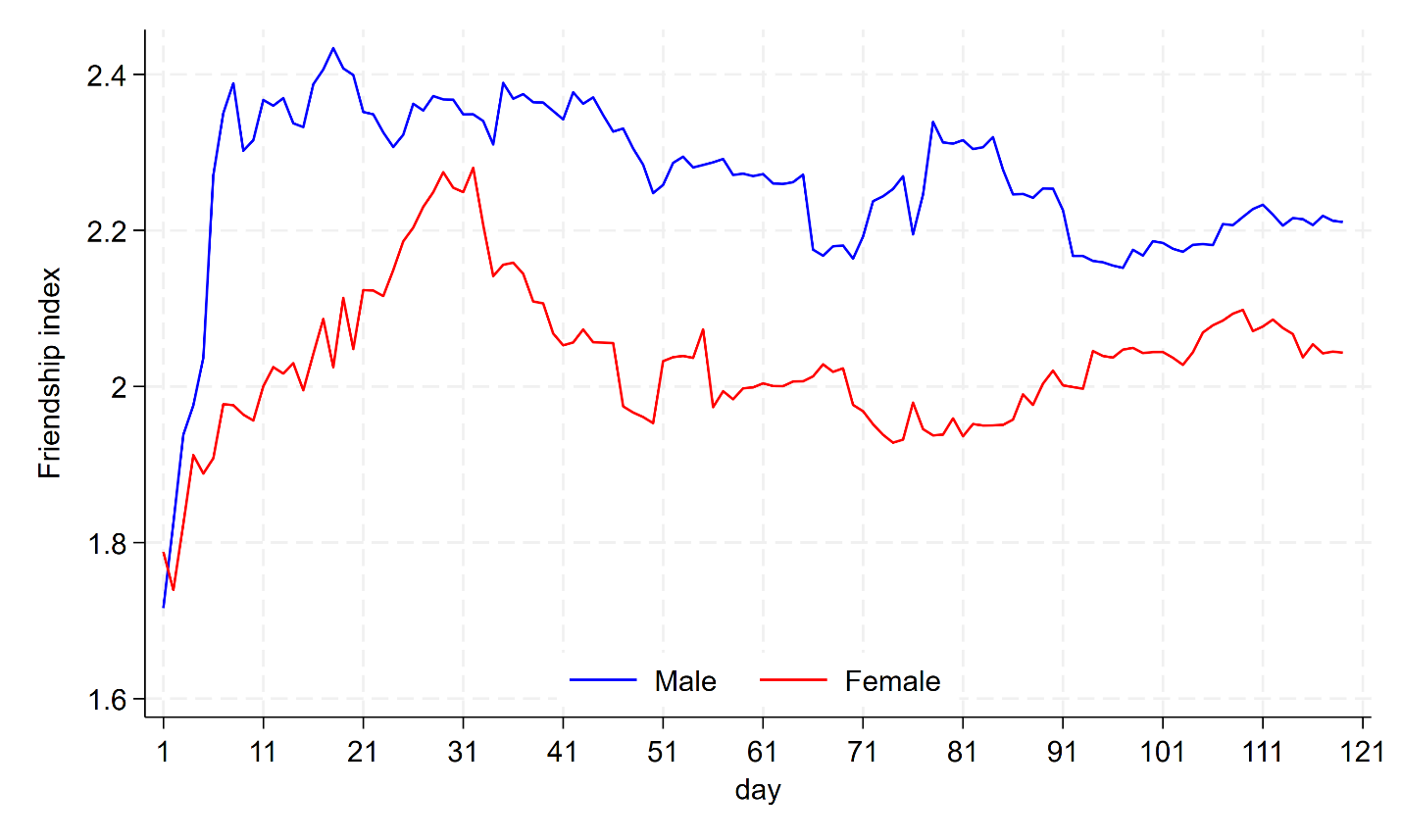


B


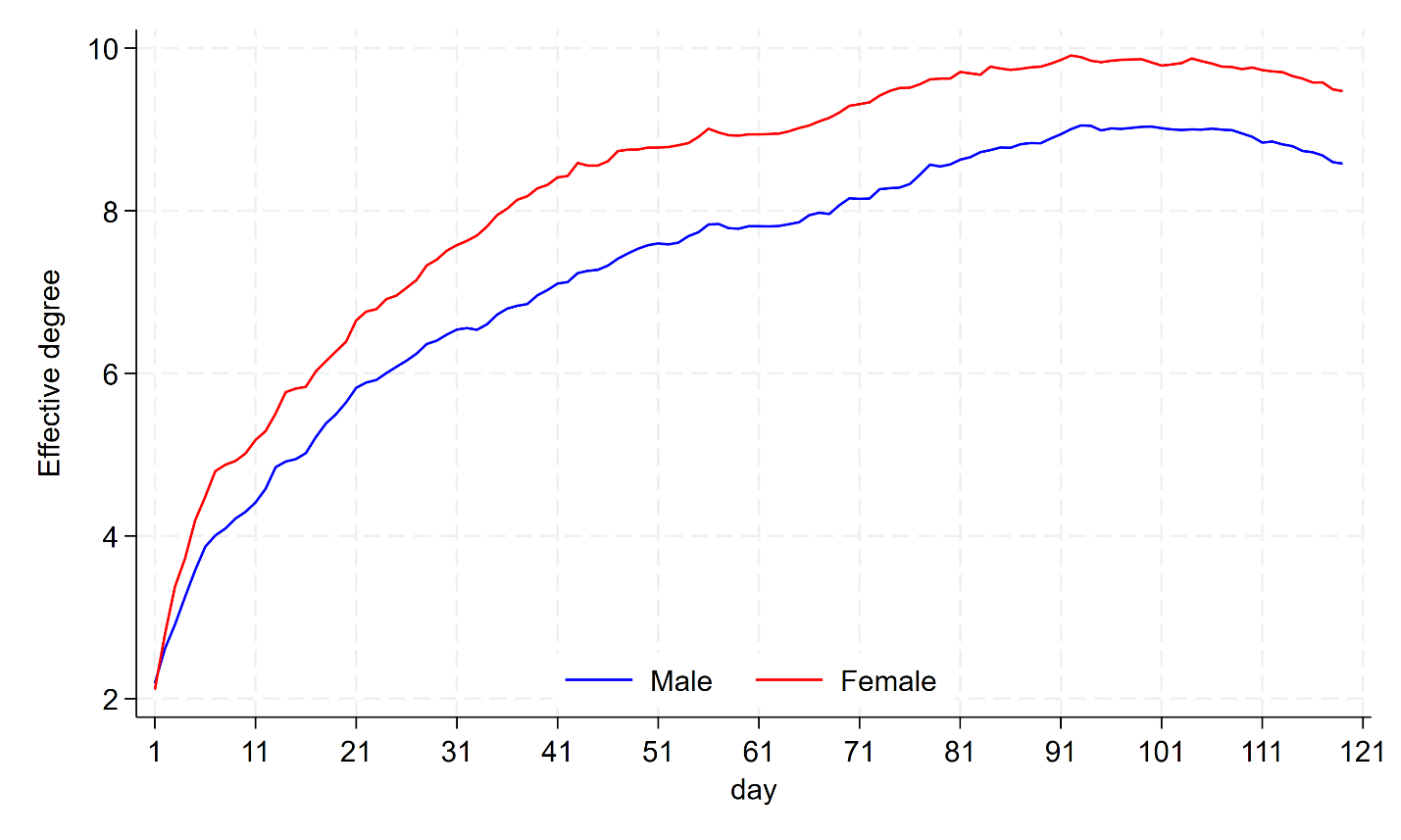


C


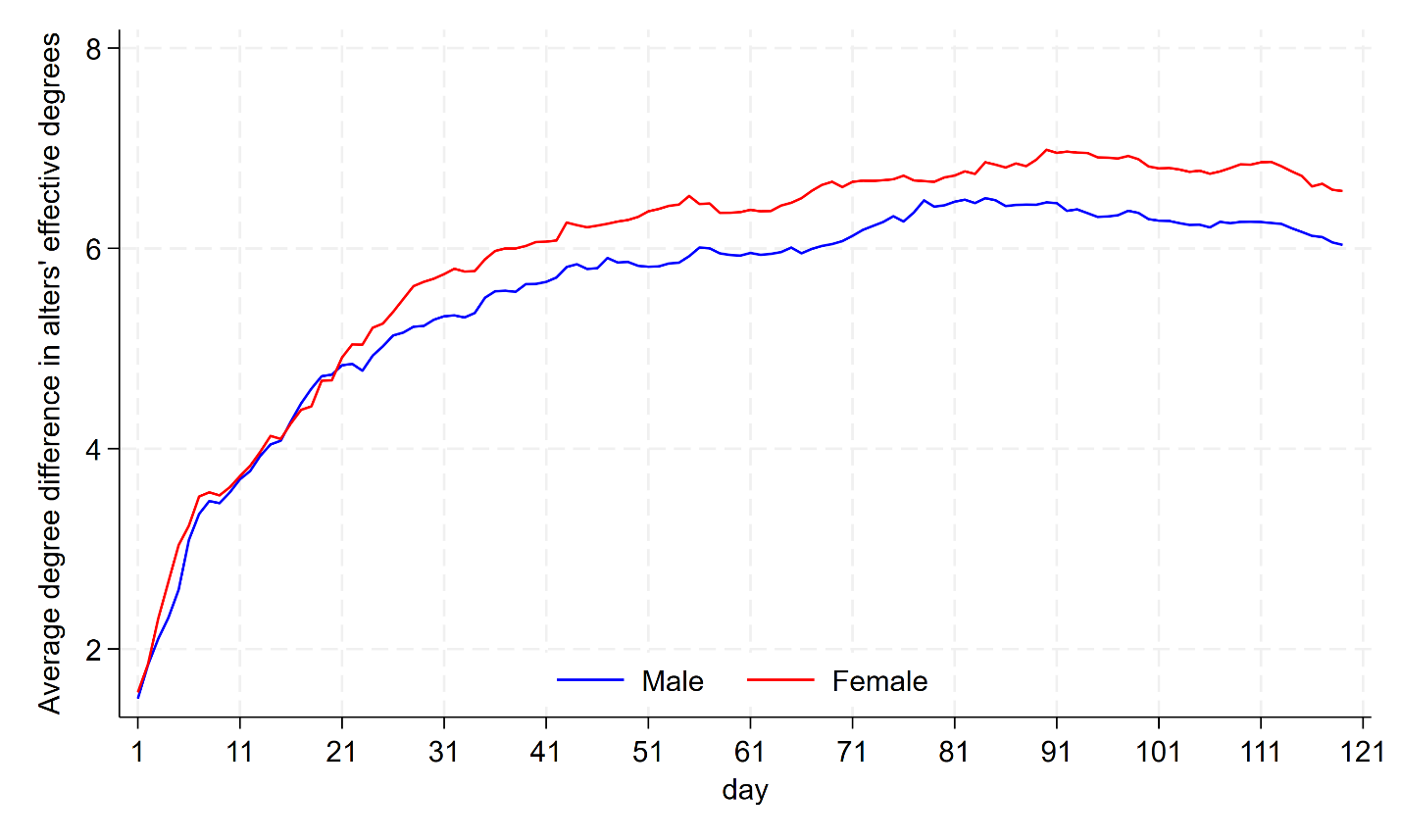


## **Fig. 6** Temporal trend of friendship index, effective degree, and average degree difference in alters’ effective degrees by gender from 08/23/2015 to 12/19/2015

A


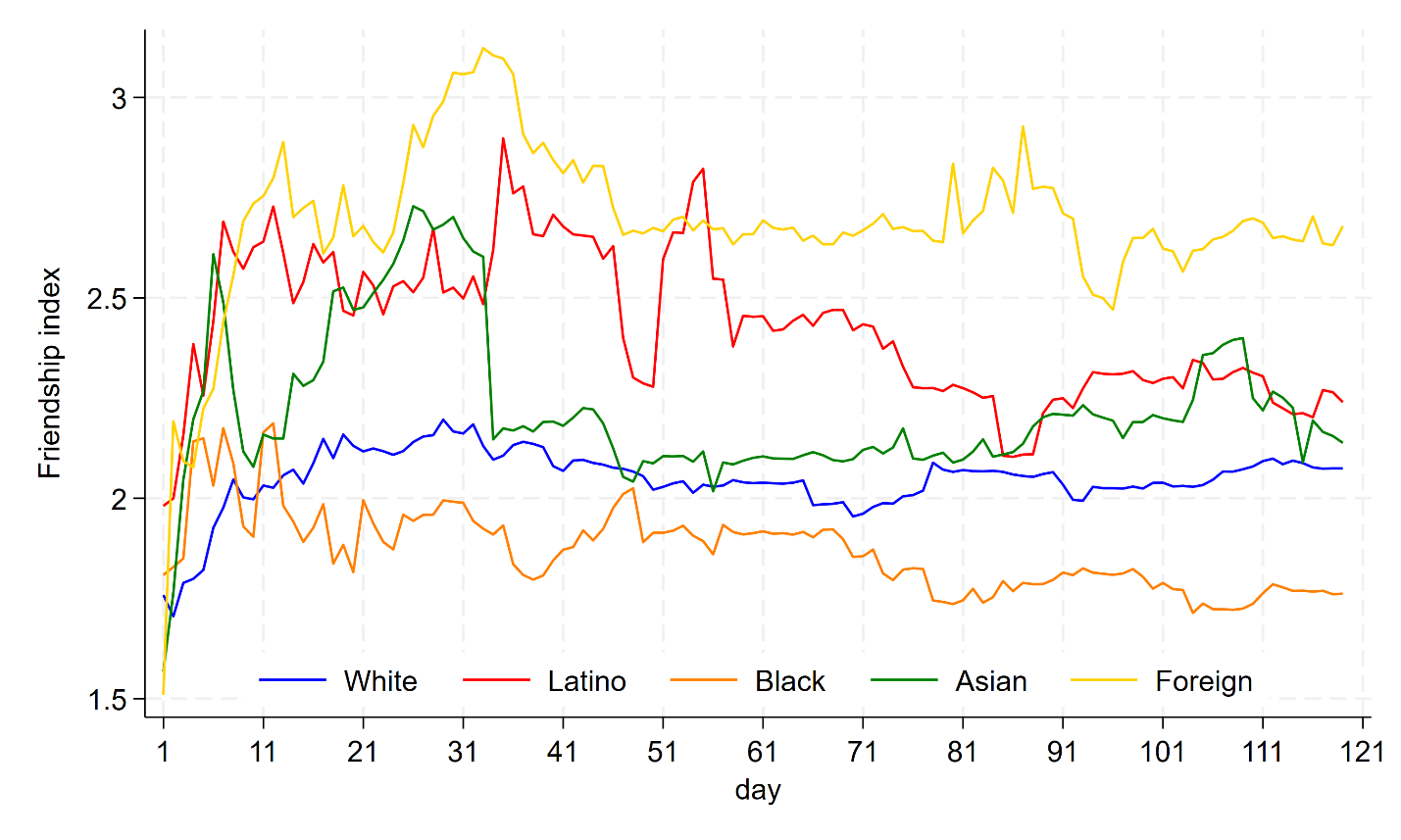


B


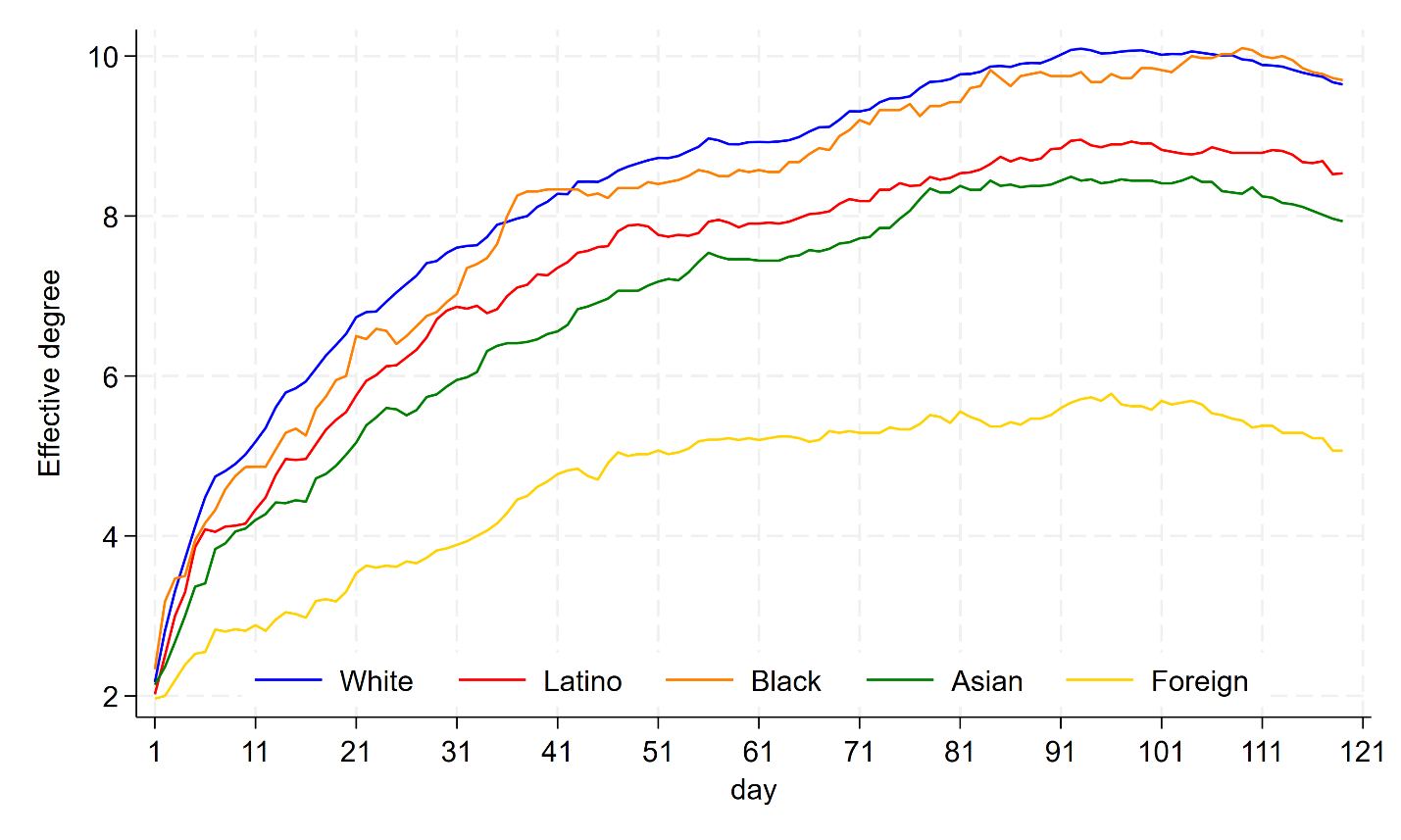


C


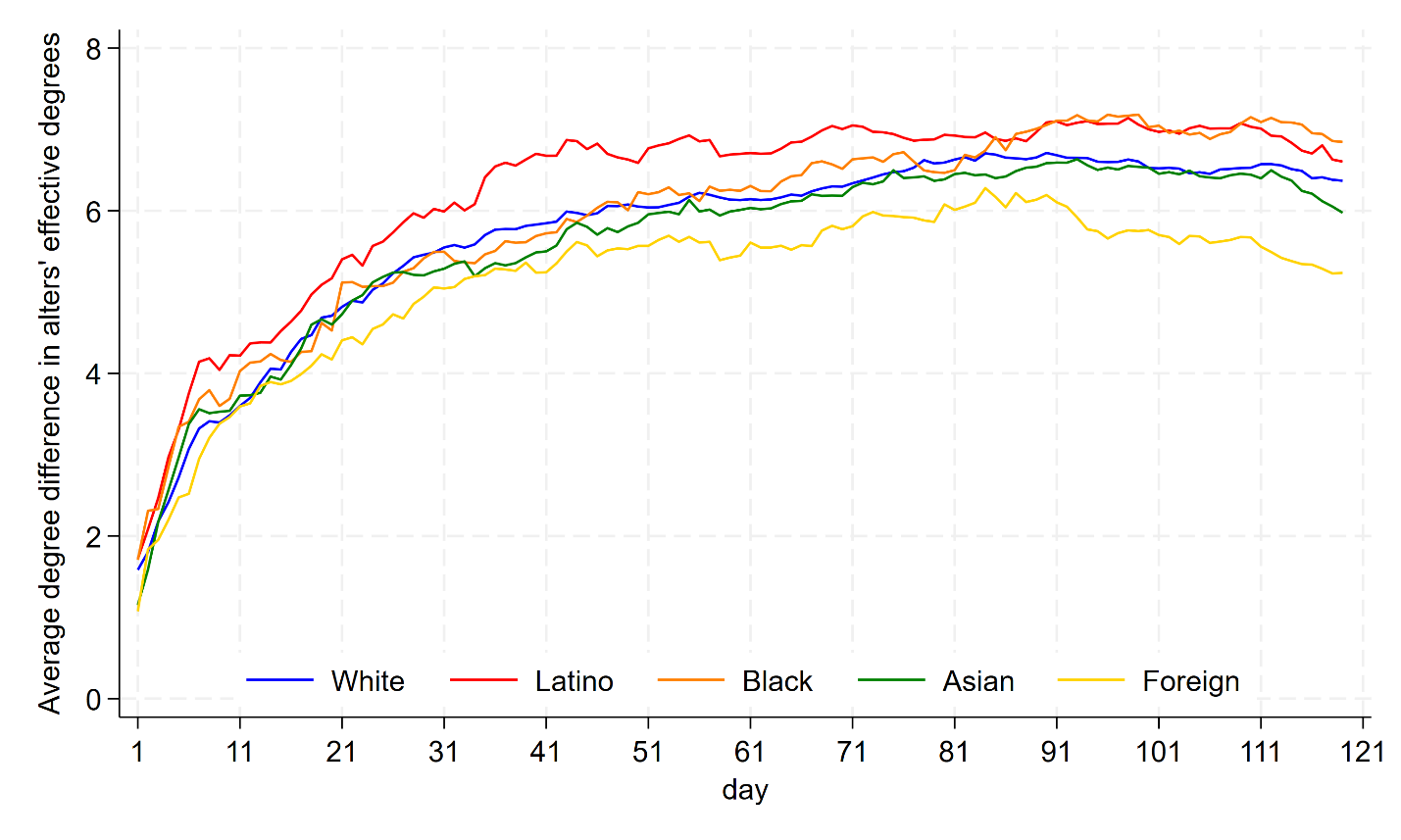


## **Fig. 7** Temporal trend of friendship index, effective degree, and average degree difference in alters’ effective degrees by race/ethnicity from 08/23/2015 to 12/19/2015

## A


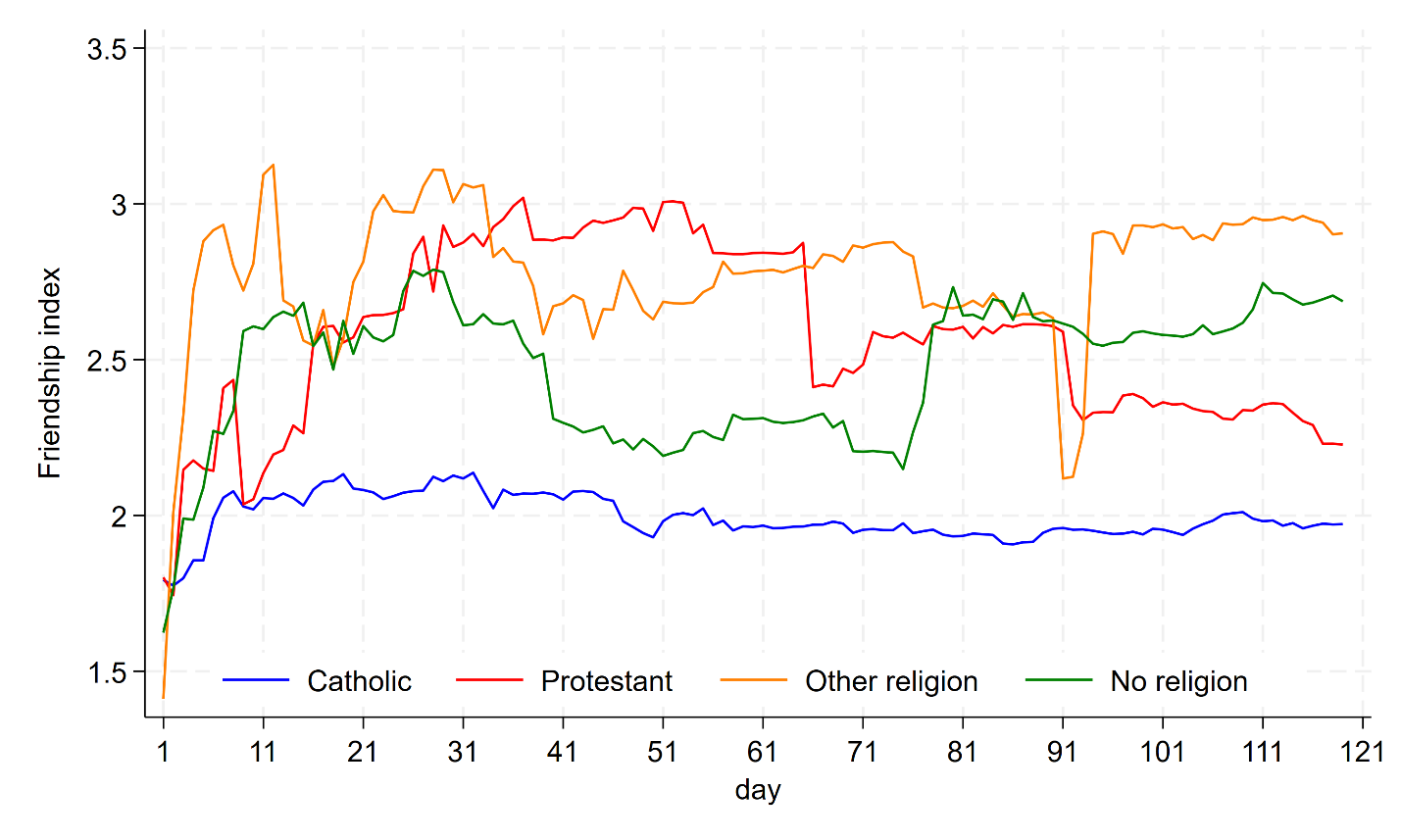


B


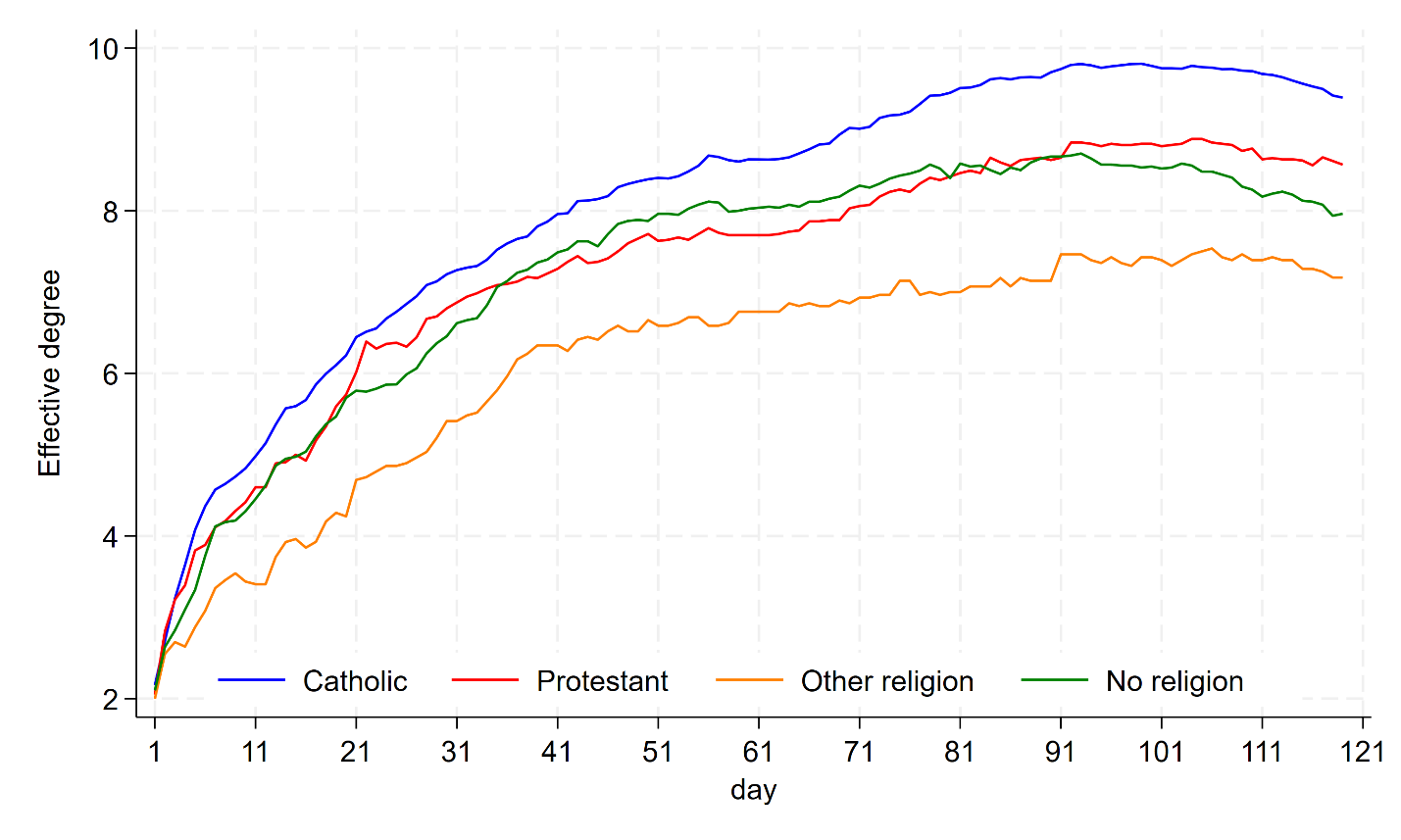


C


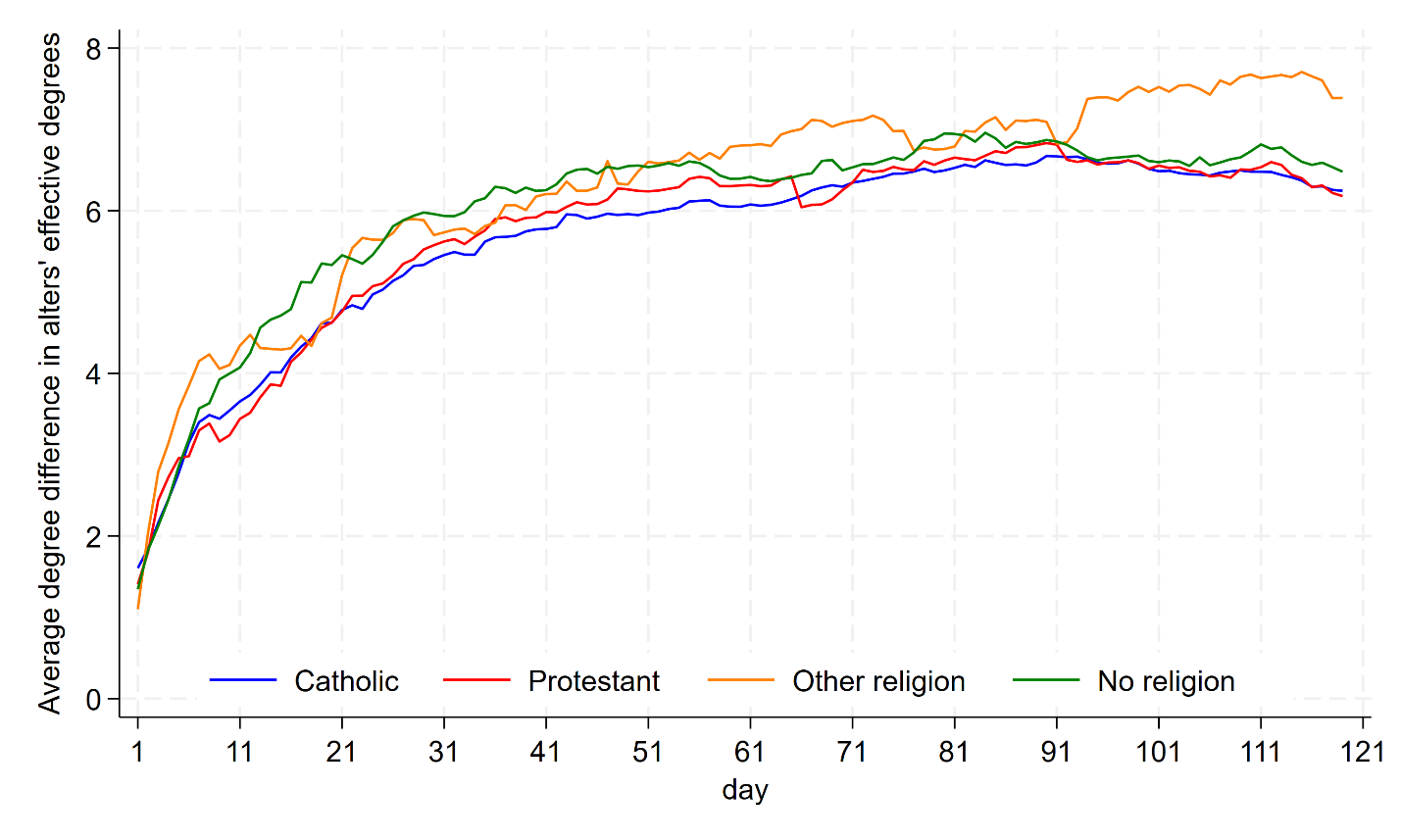


## **Fig. 8** Temporal trend of friendship index, effective degree, and average degree difference in alters’ effective degrees by religious preference from 08/23/2015 to 12/19/2015

A


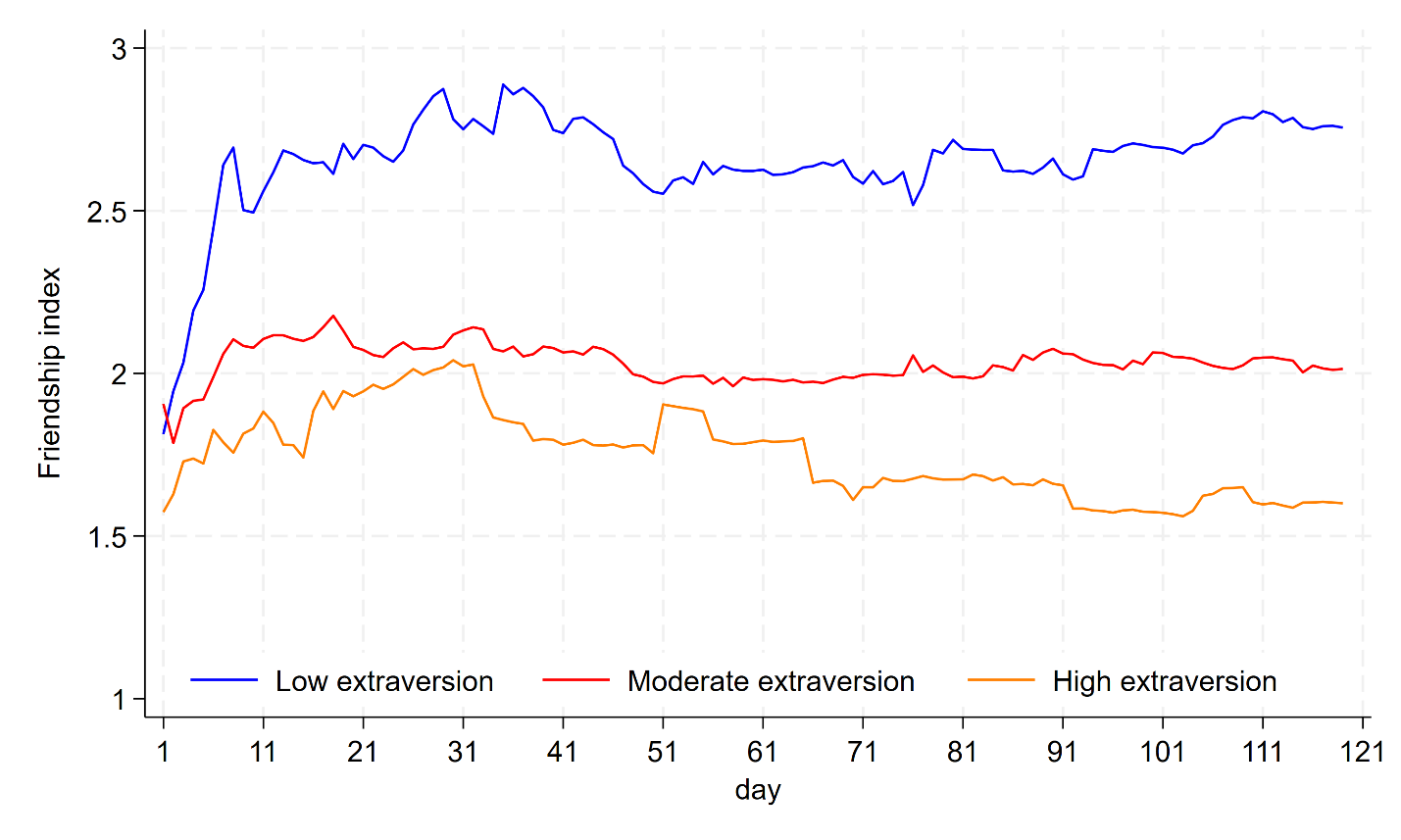


B


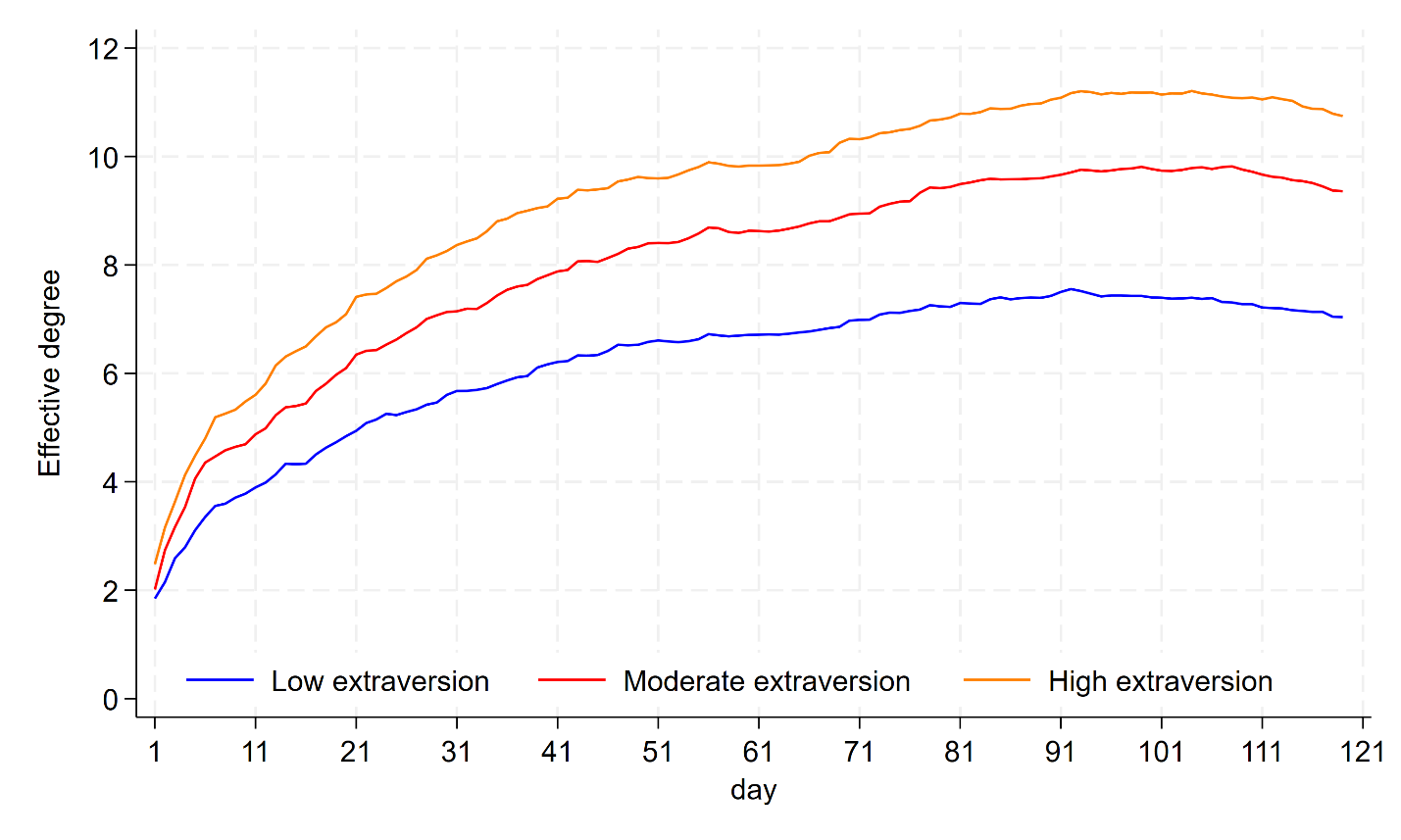


C


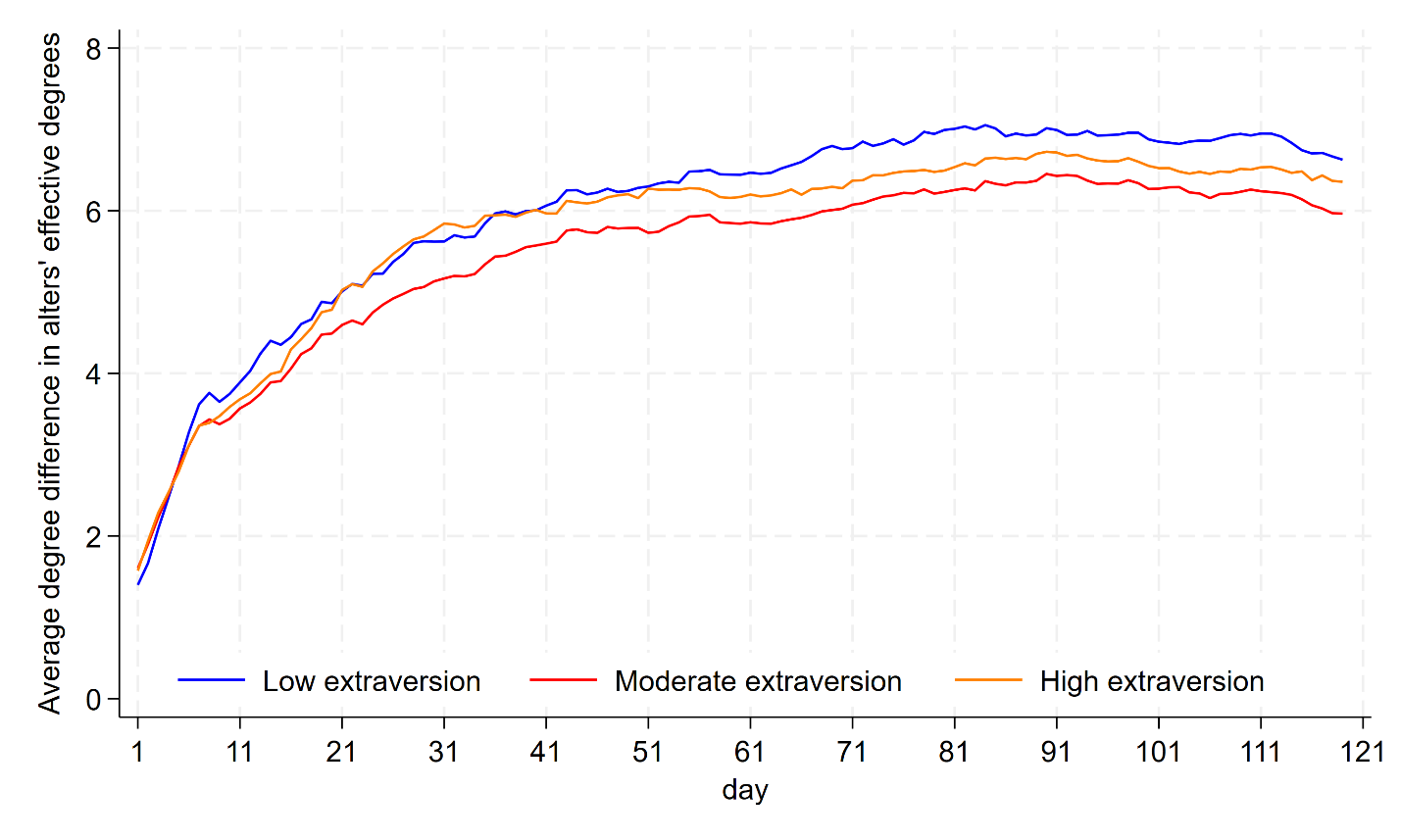


## **Fig. 9** Temporal trend of friendship index, effective degree, and average degree difference in alters’ effective degrees by extraversion from 08/23/2015 to 12/19/2015

## A


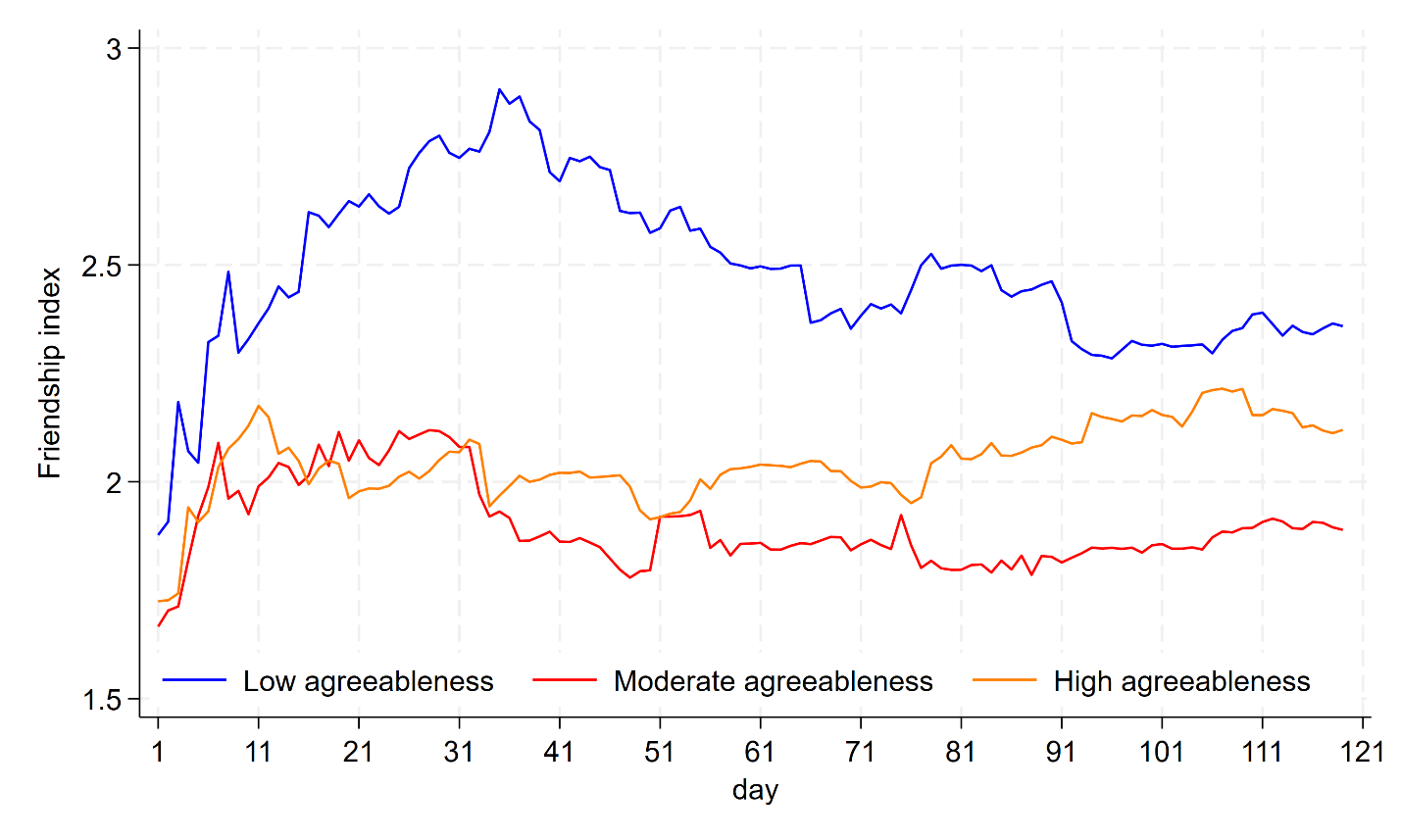


B


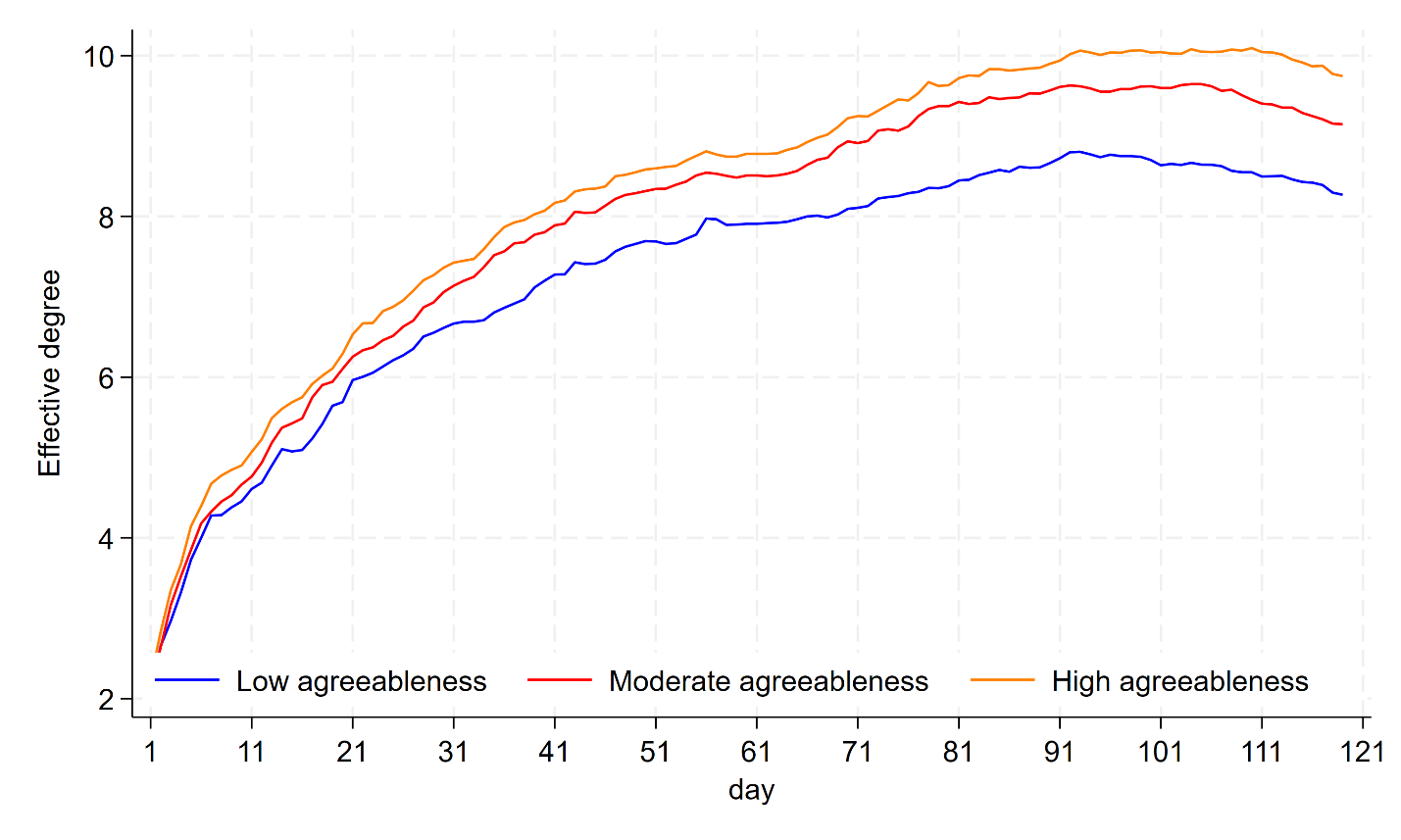


C


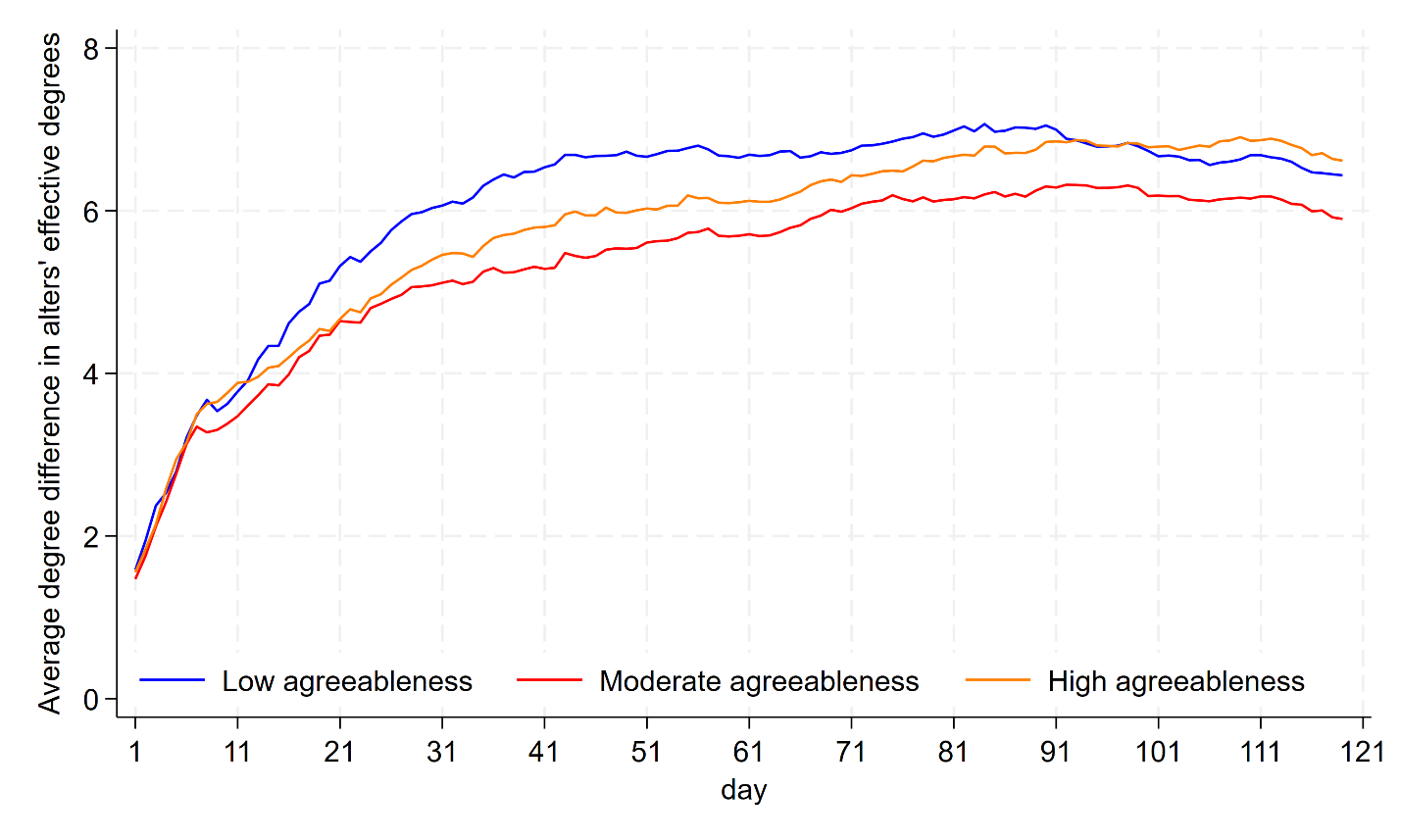


## **Fig. 10** Temporal trend of friendship index, effective degree, and average degree difference in alters’ effective degrees by agreeableness from 08/23/2015 to 12/19/2015

## A


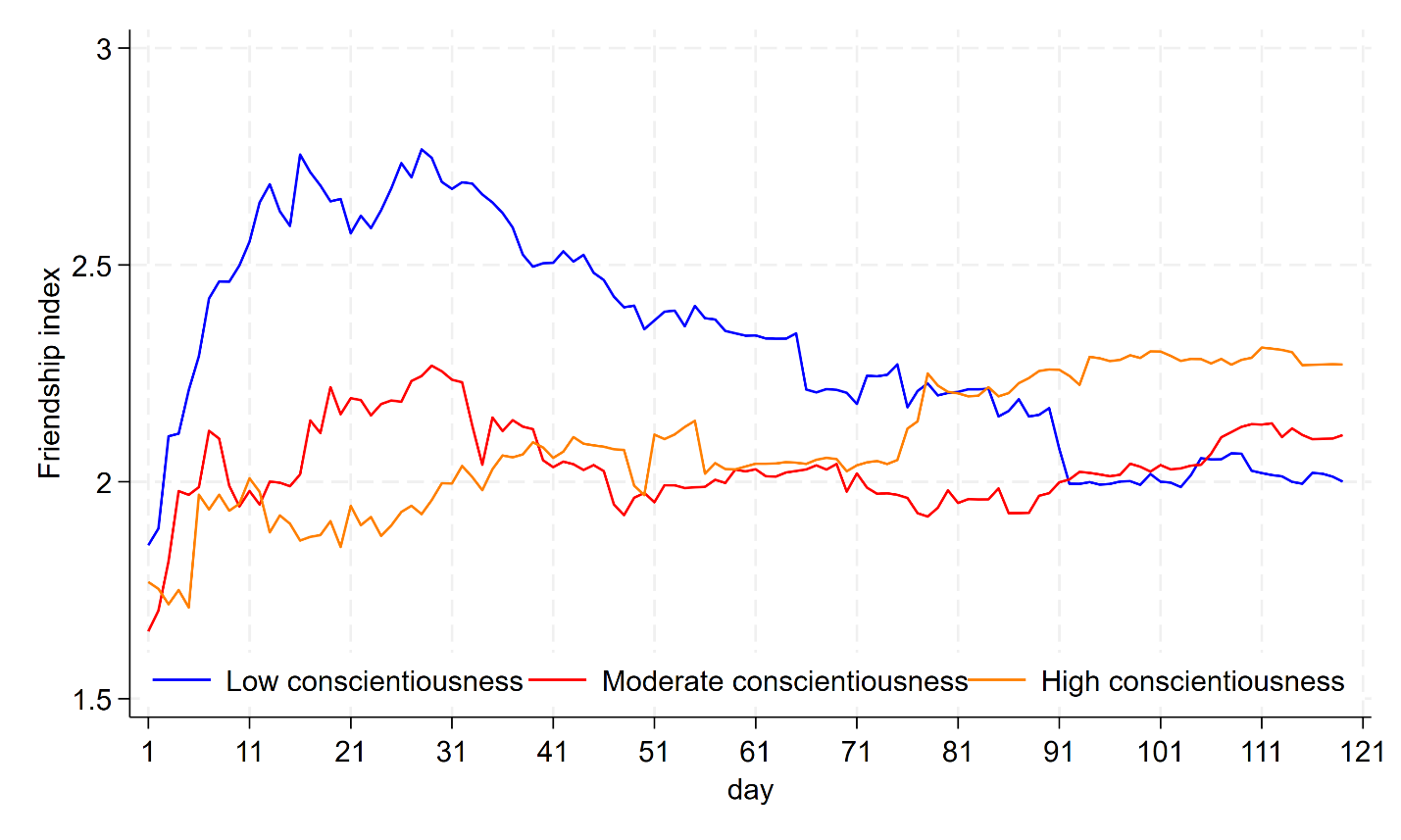


B


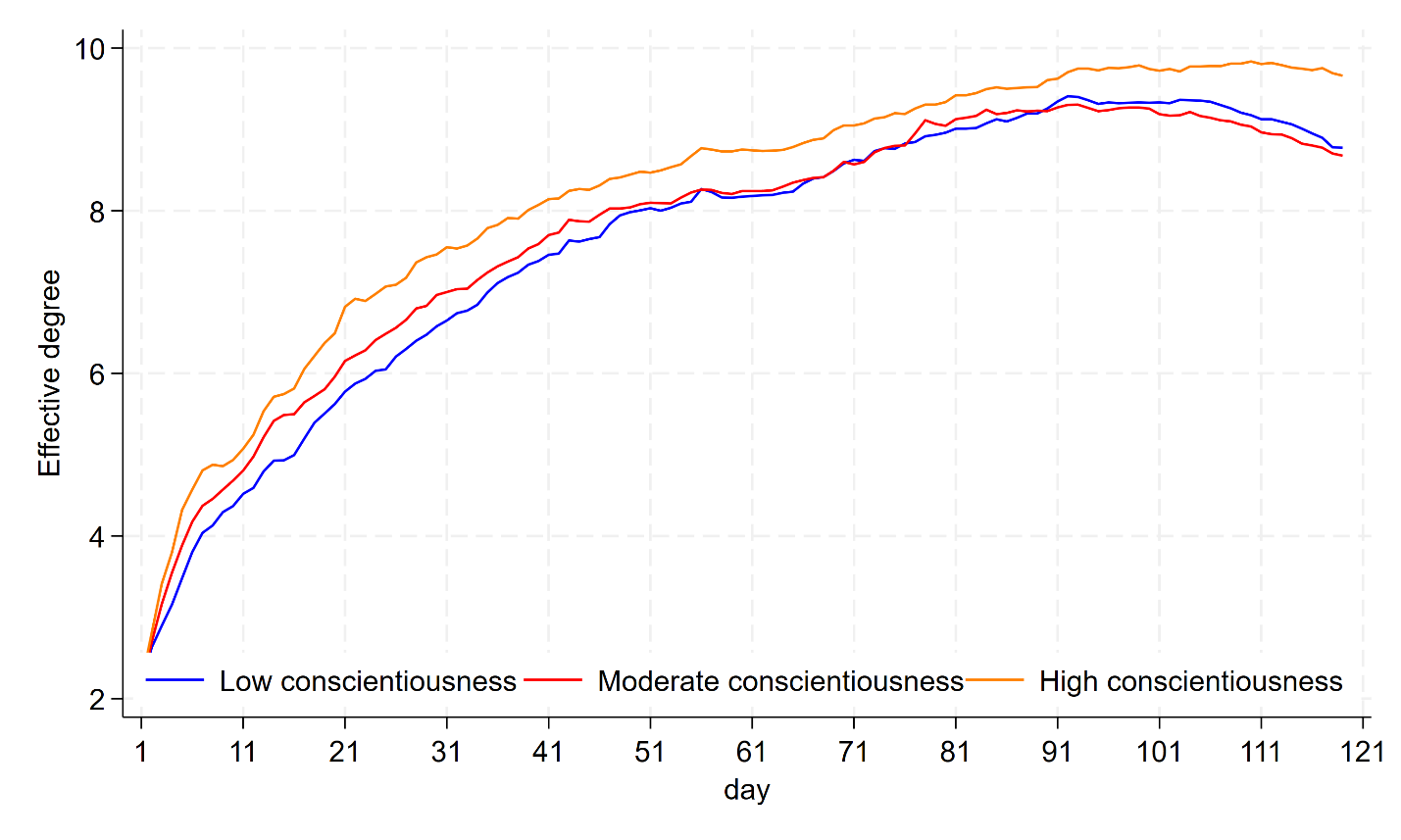


C


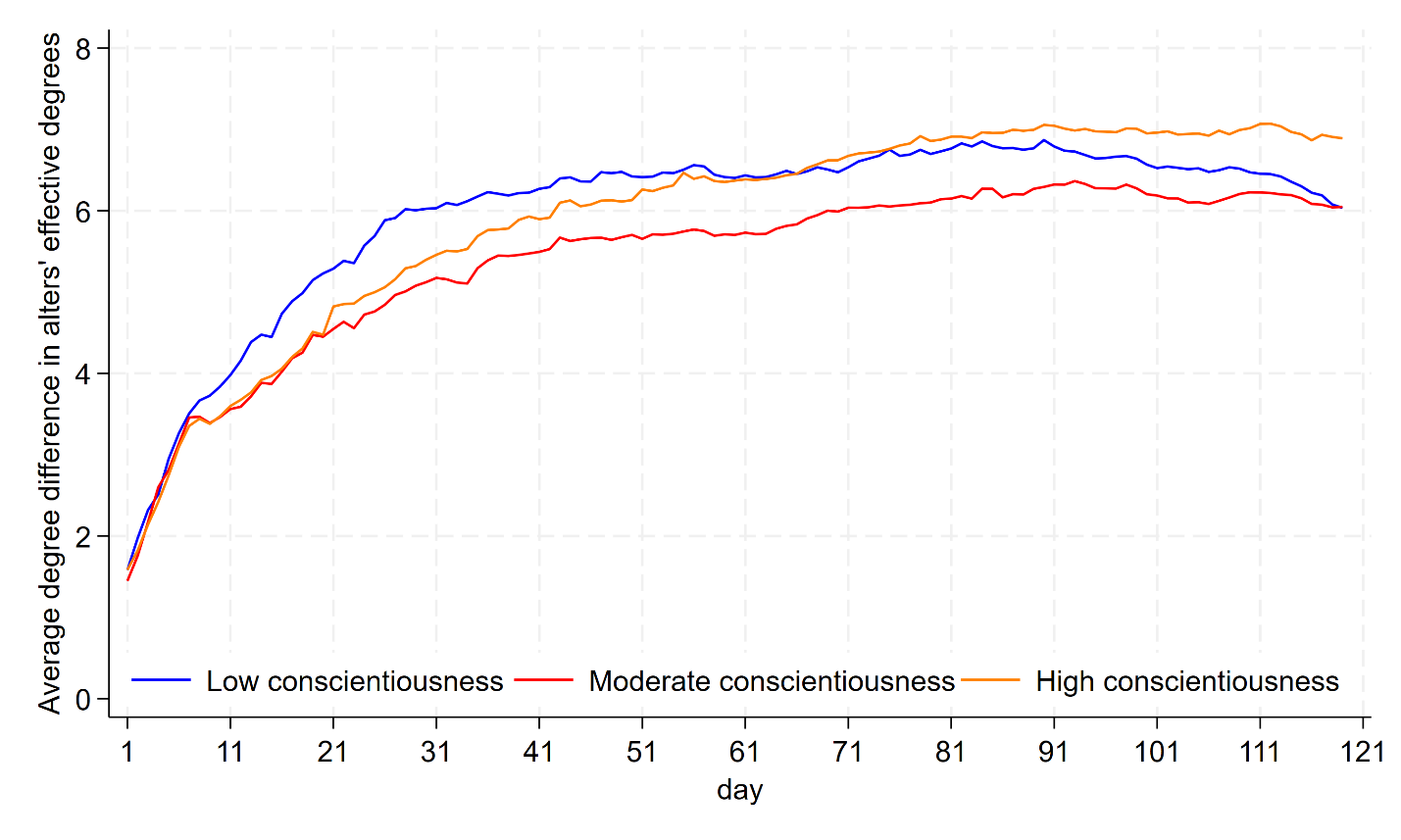


## **Fig. 11** Temporal trend of friendship index, effective degree, and average degree difference in alters’ effective degrees by conscientiousness from 08/23/2015 to 12/19/2015

A


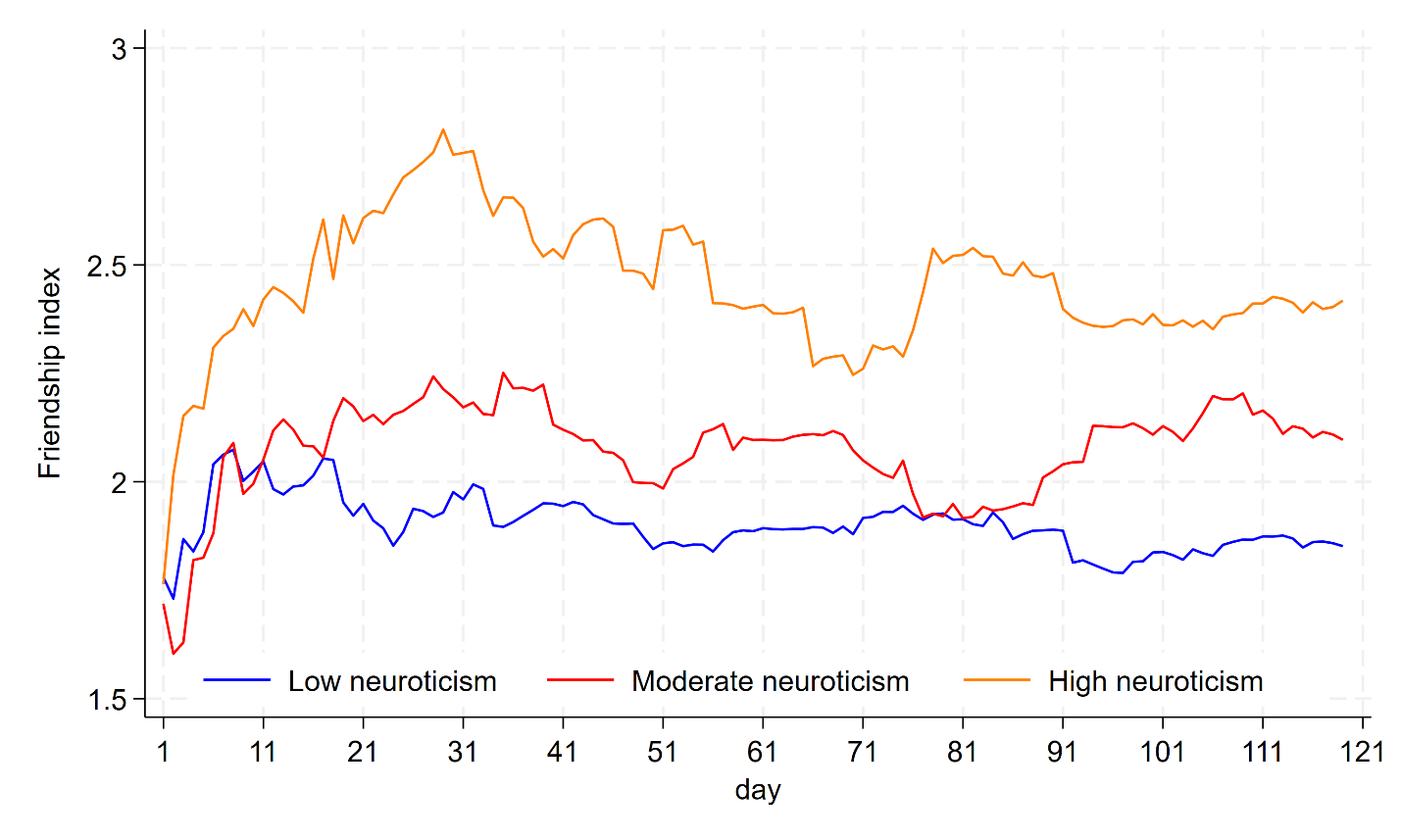


B


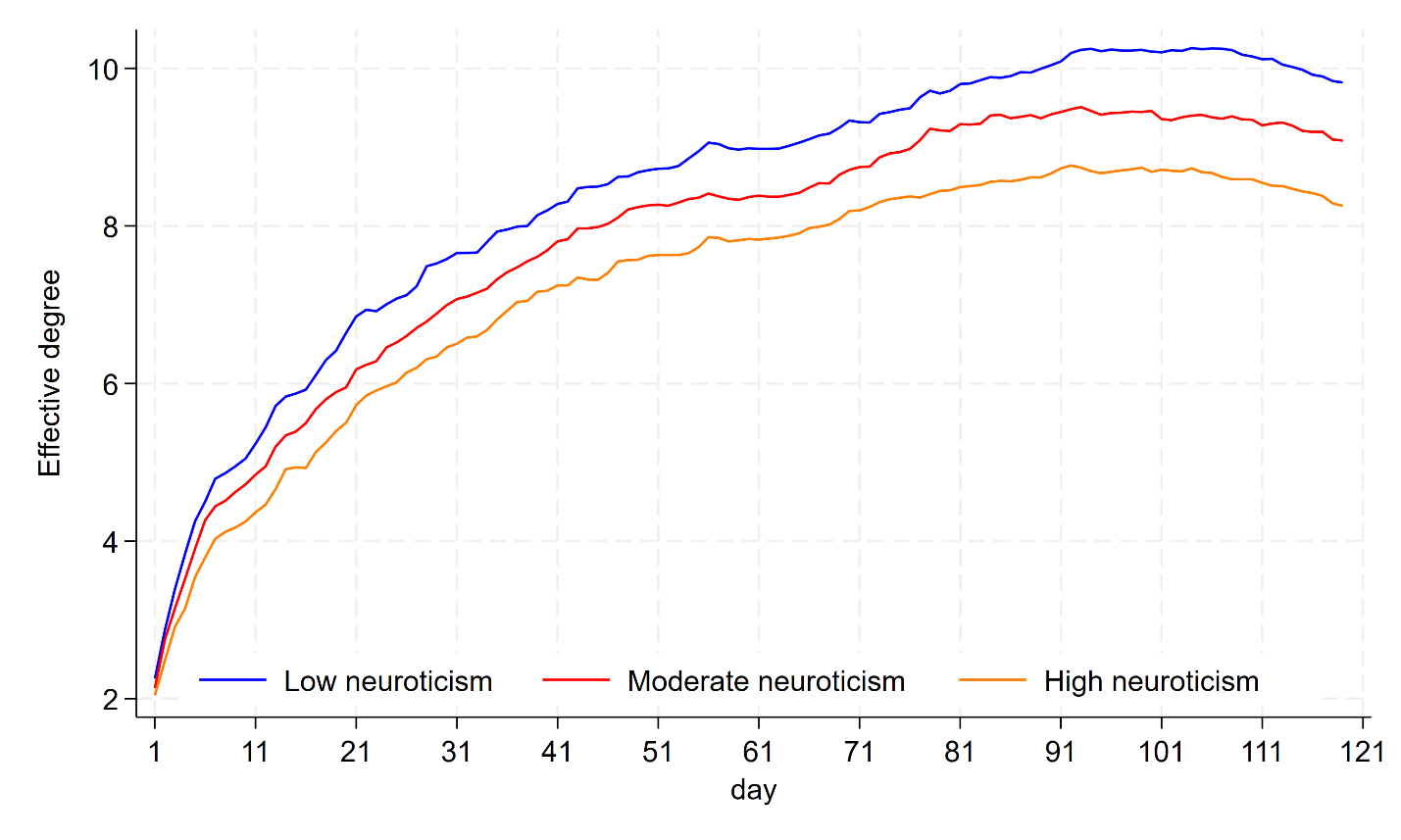


C


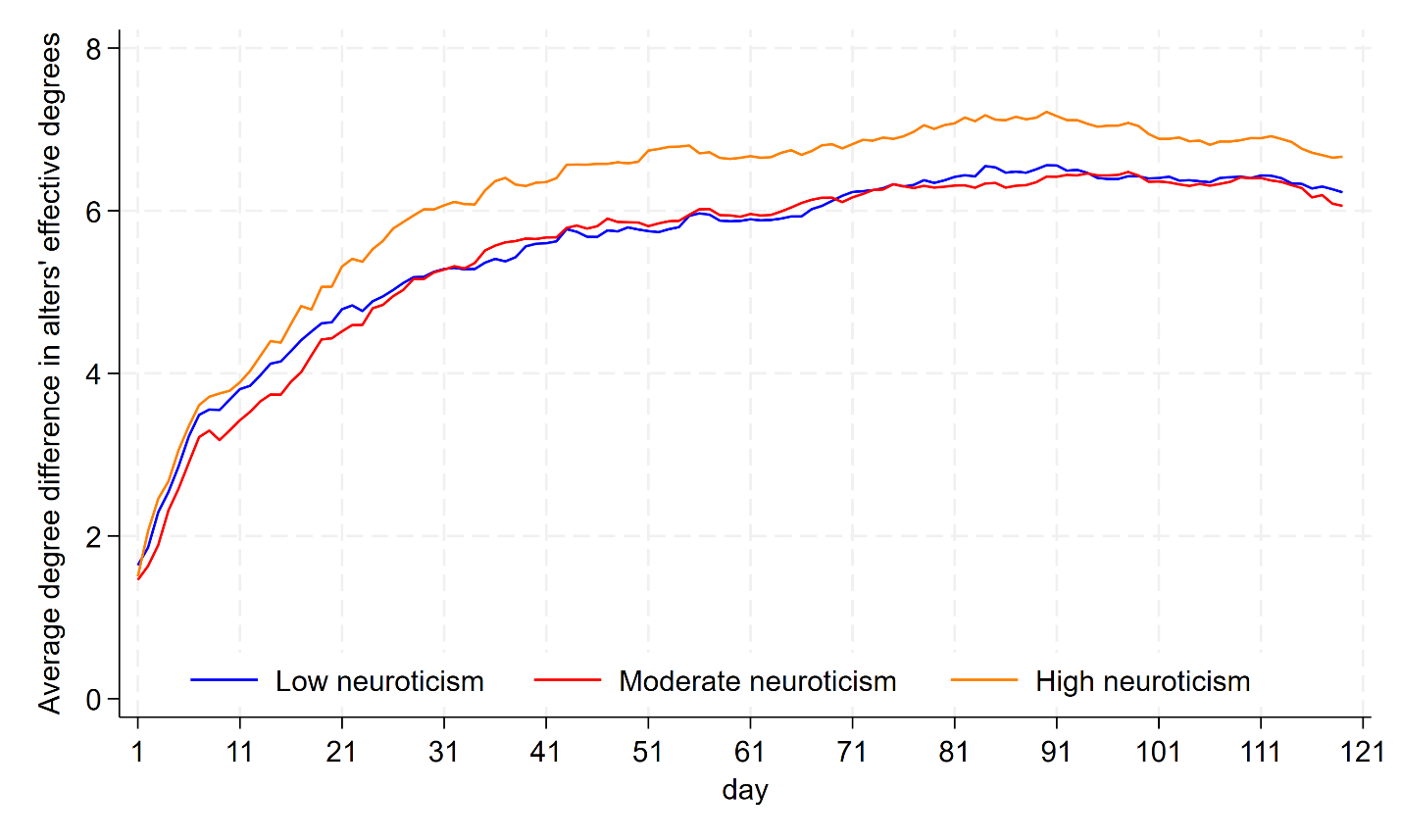


## **Fig. 12** Temporal trend of friendship index, effective degree, and average degree difference in alters’ effective degrees by neuroticism from 08/23/2015 to 12/19/2015

A


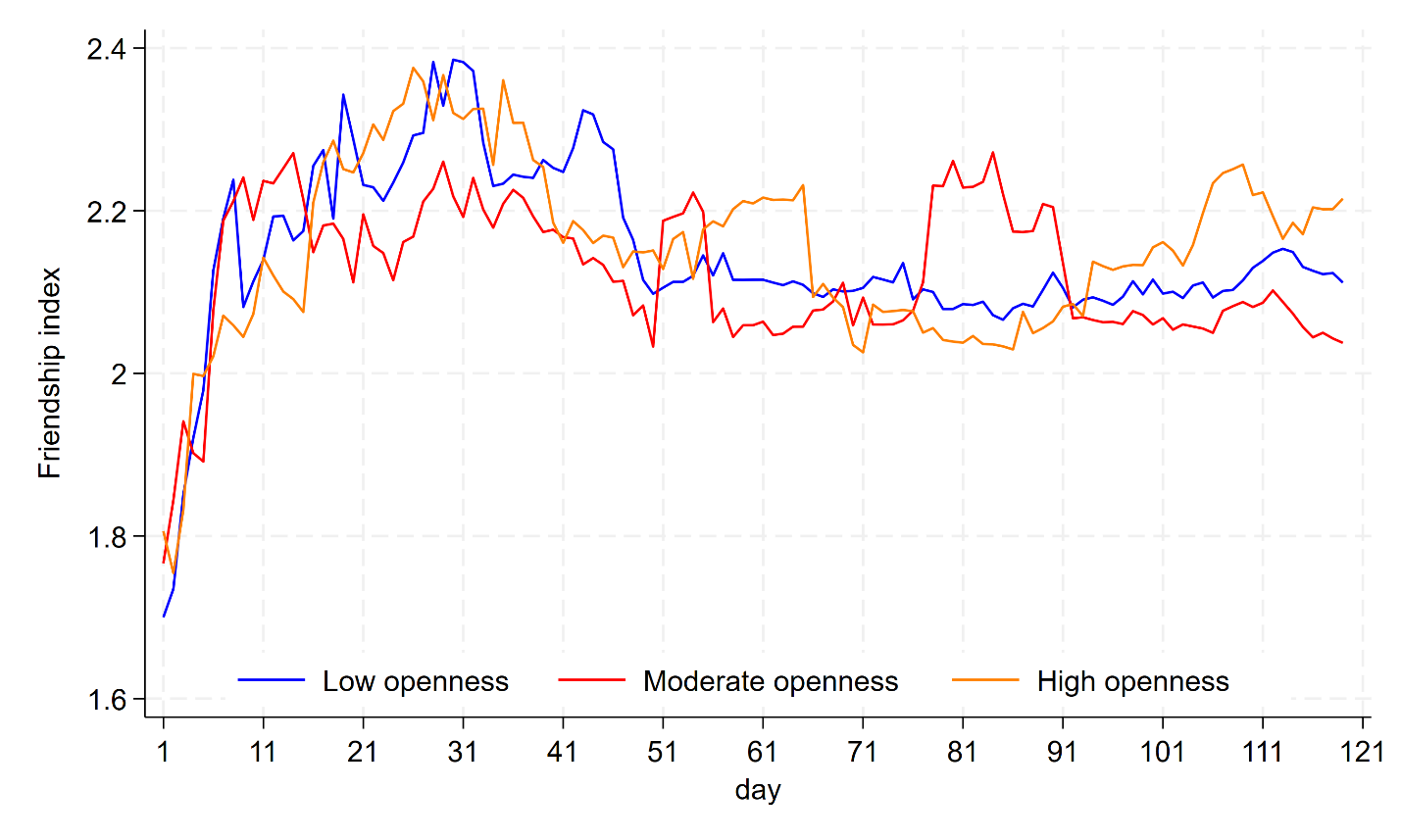


B


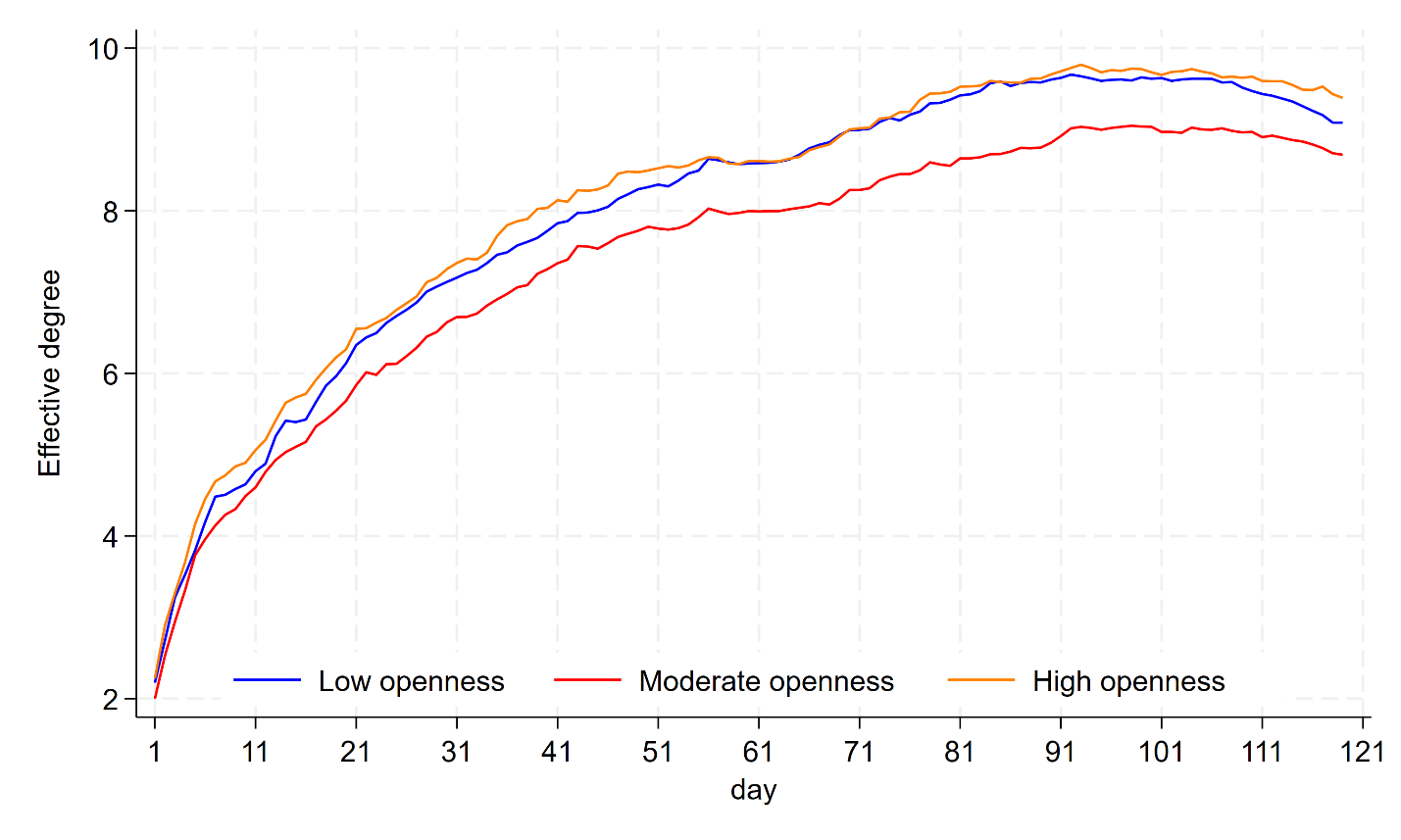


C


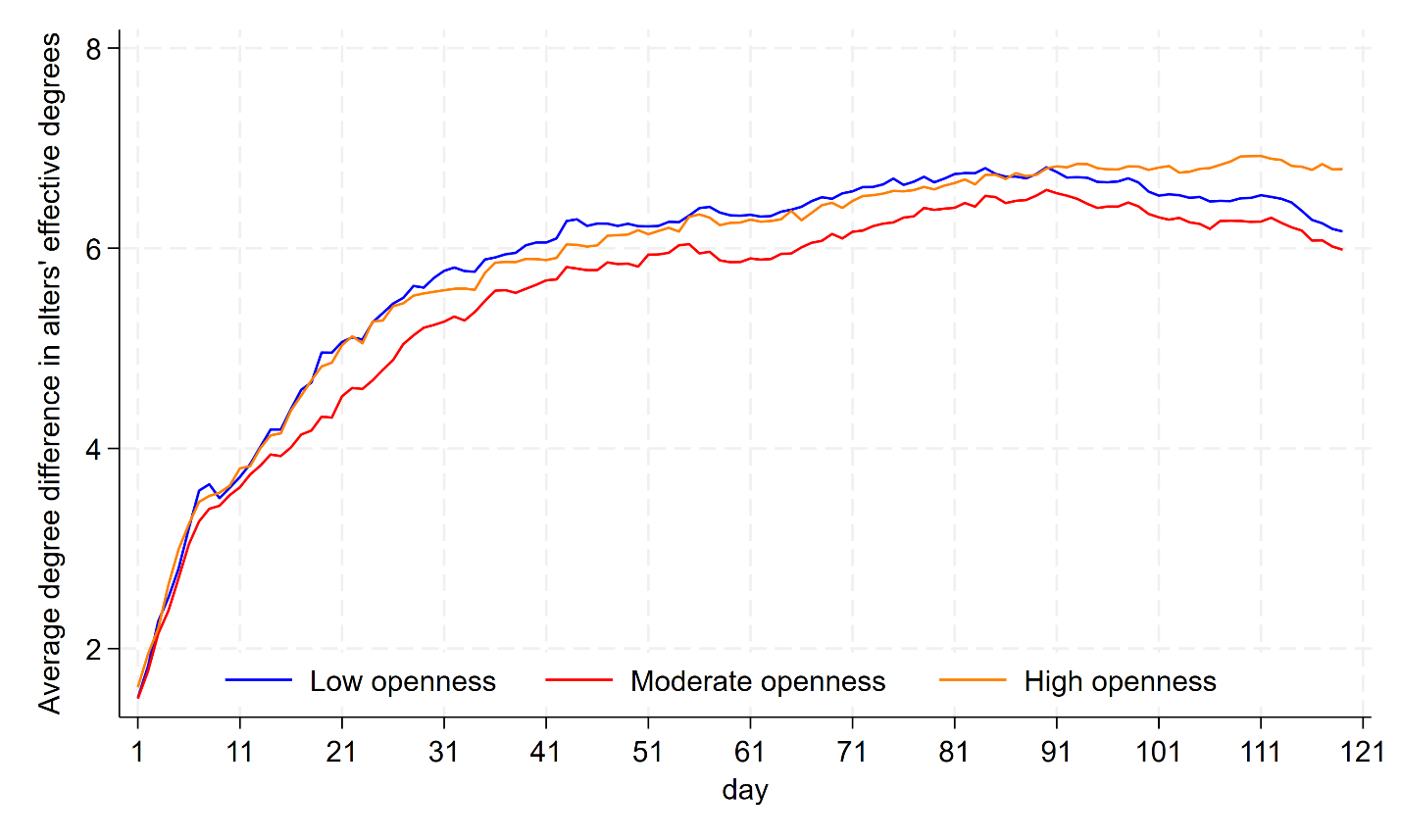


## **Fig. 13** Temporal trend of friendship index, effective degree, and average degree difference in alters’ effective degrees by openness from 08/23/2015 to 12/19/2015

## A


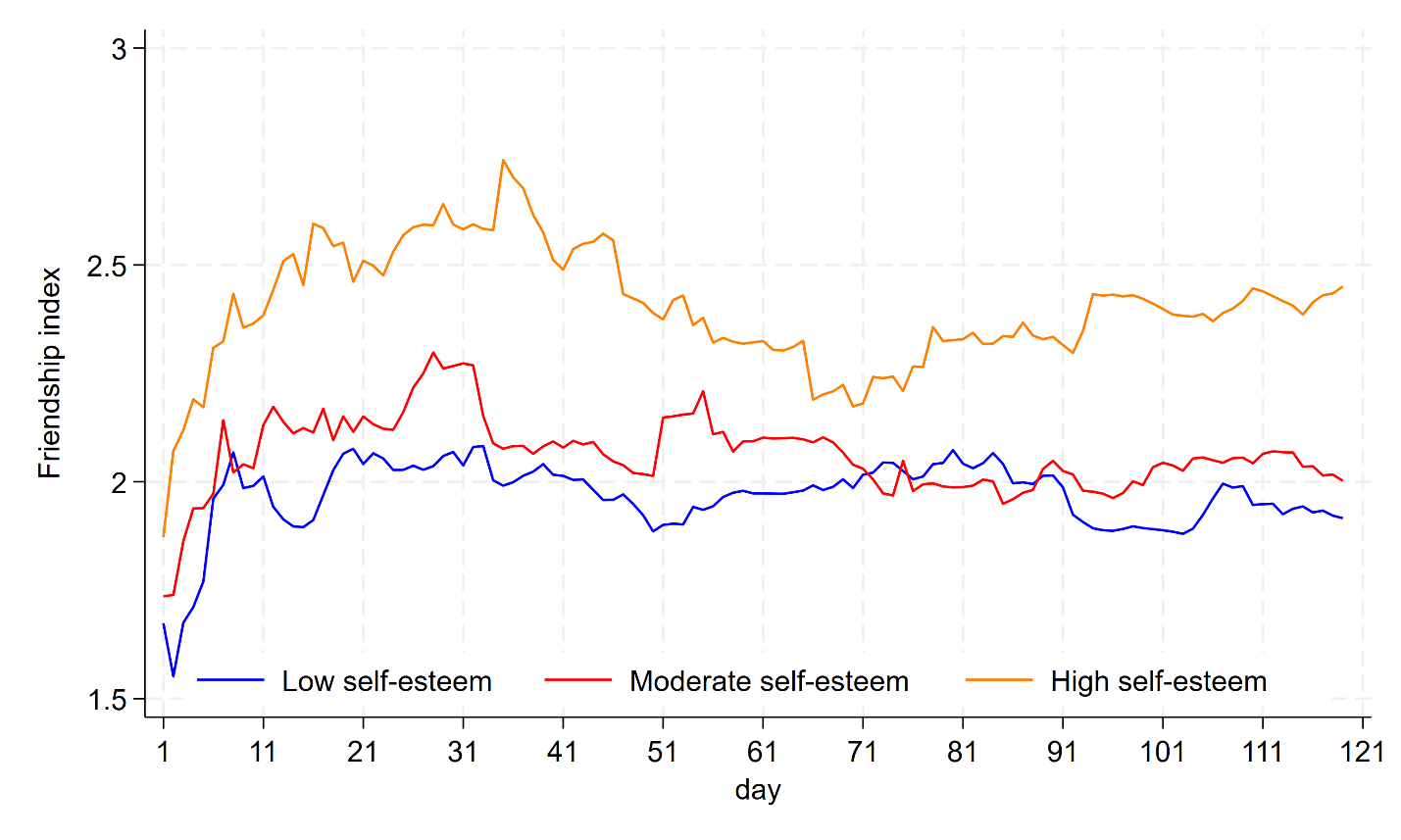


B


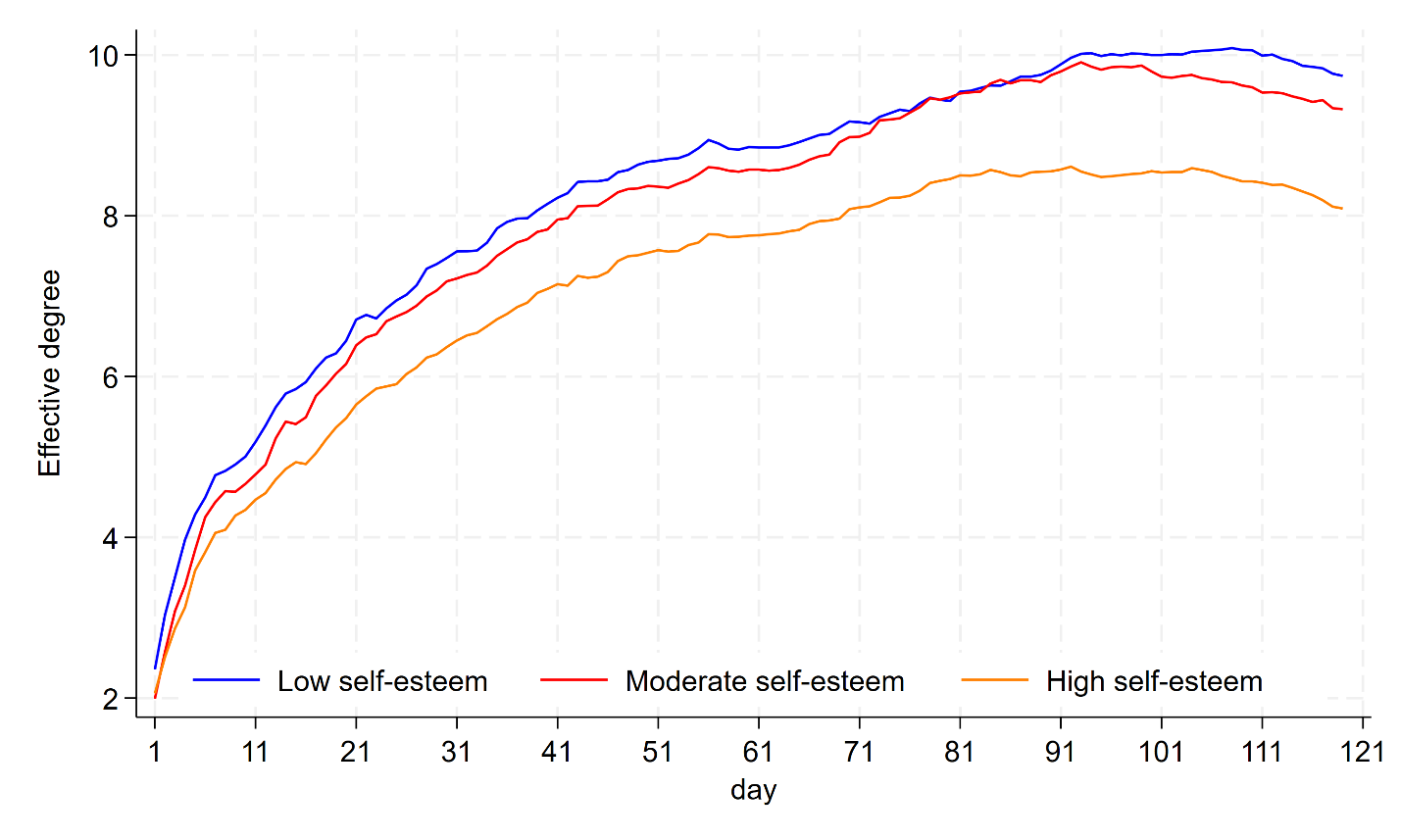


C


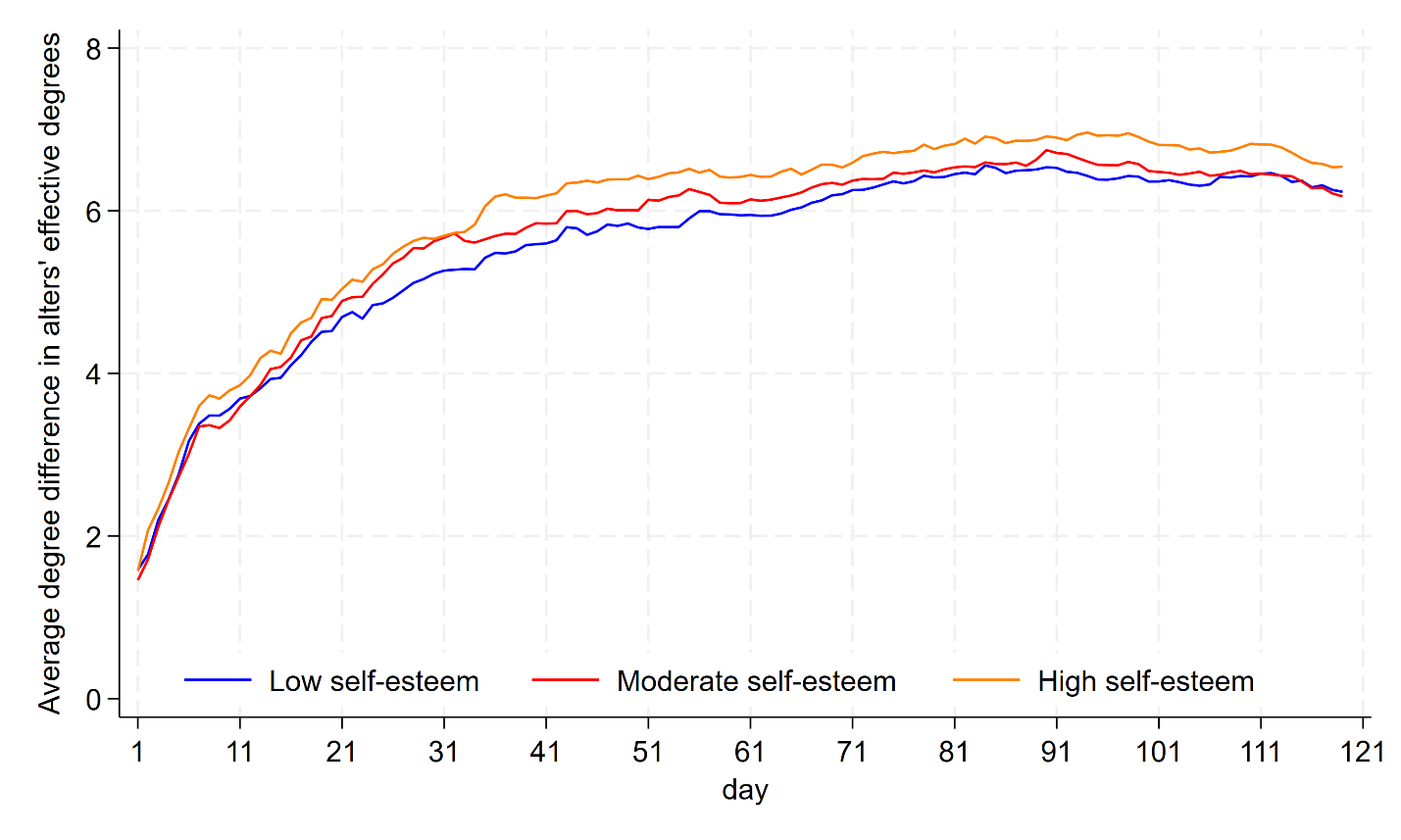


## **Fig. 14** Temporal trend of friendship index, effective degree, and average degree difference in alters’ effective degrees by self-esteem from 08/23/2015 to 12/19/2015

A


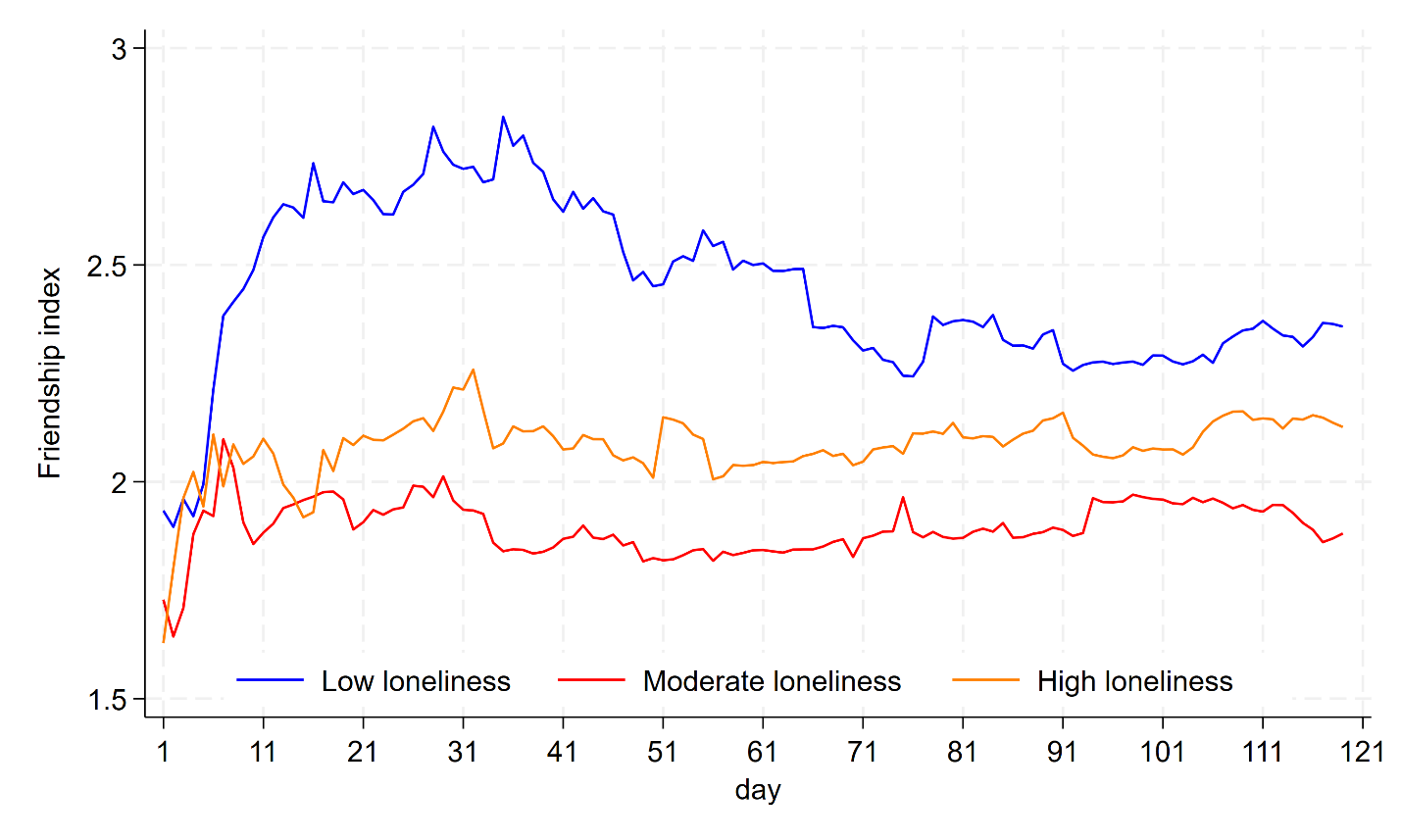


B


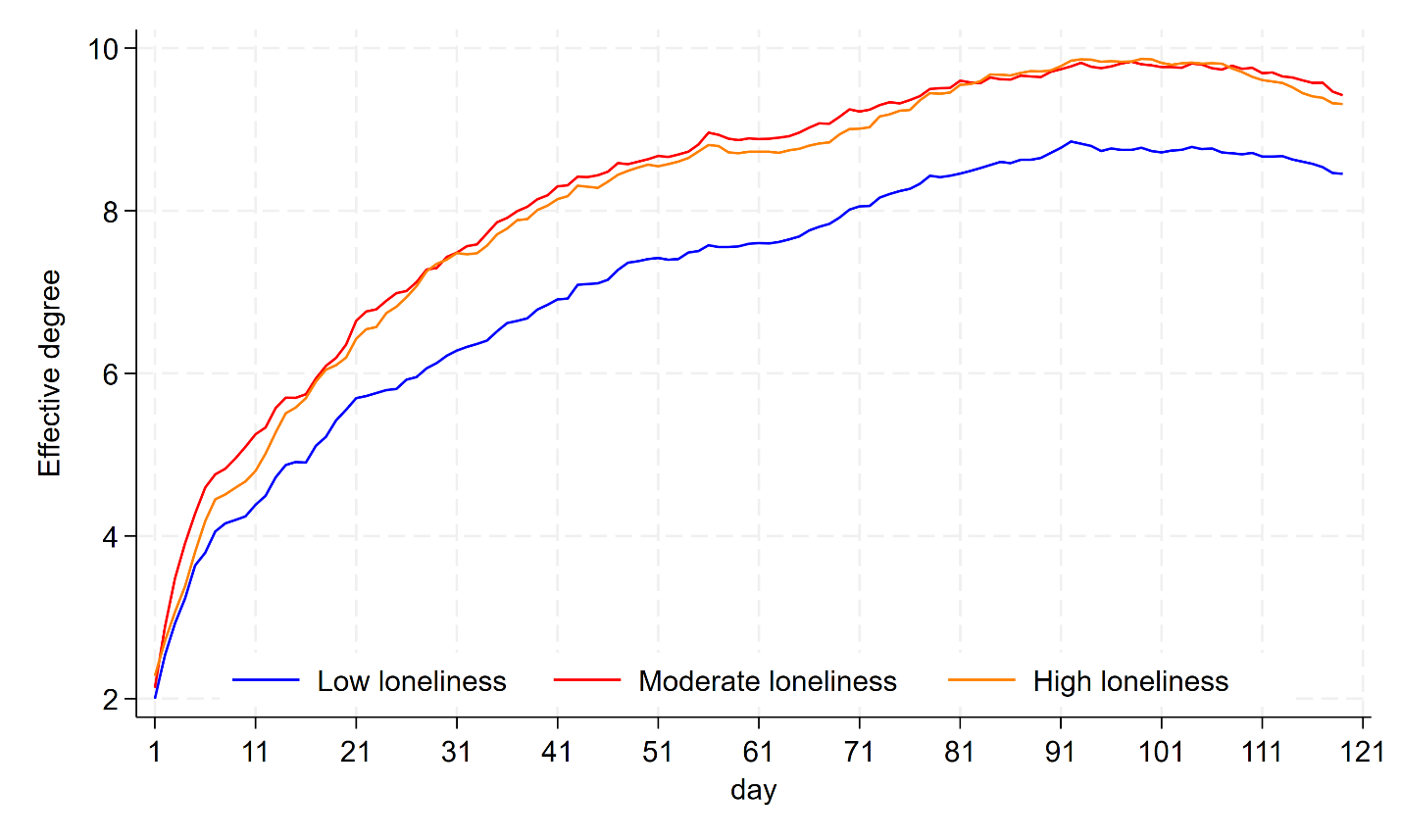


C


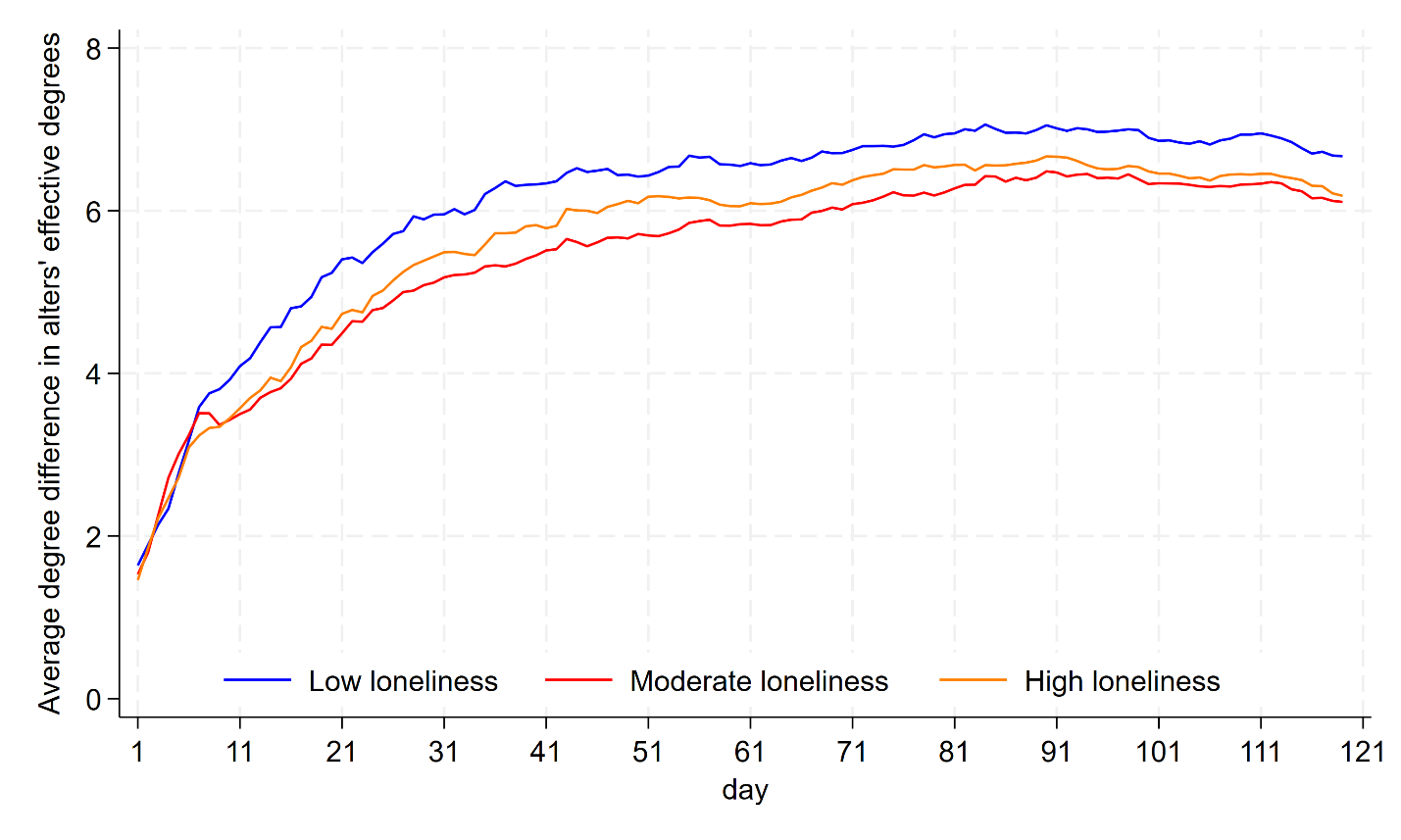


## **Fig. 15** Temporal trend of friendship index, effective degree, and average degree difference in alters’ effective degrees by loneliness from 08/23/2015 to 12/19/2015

A


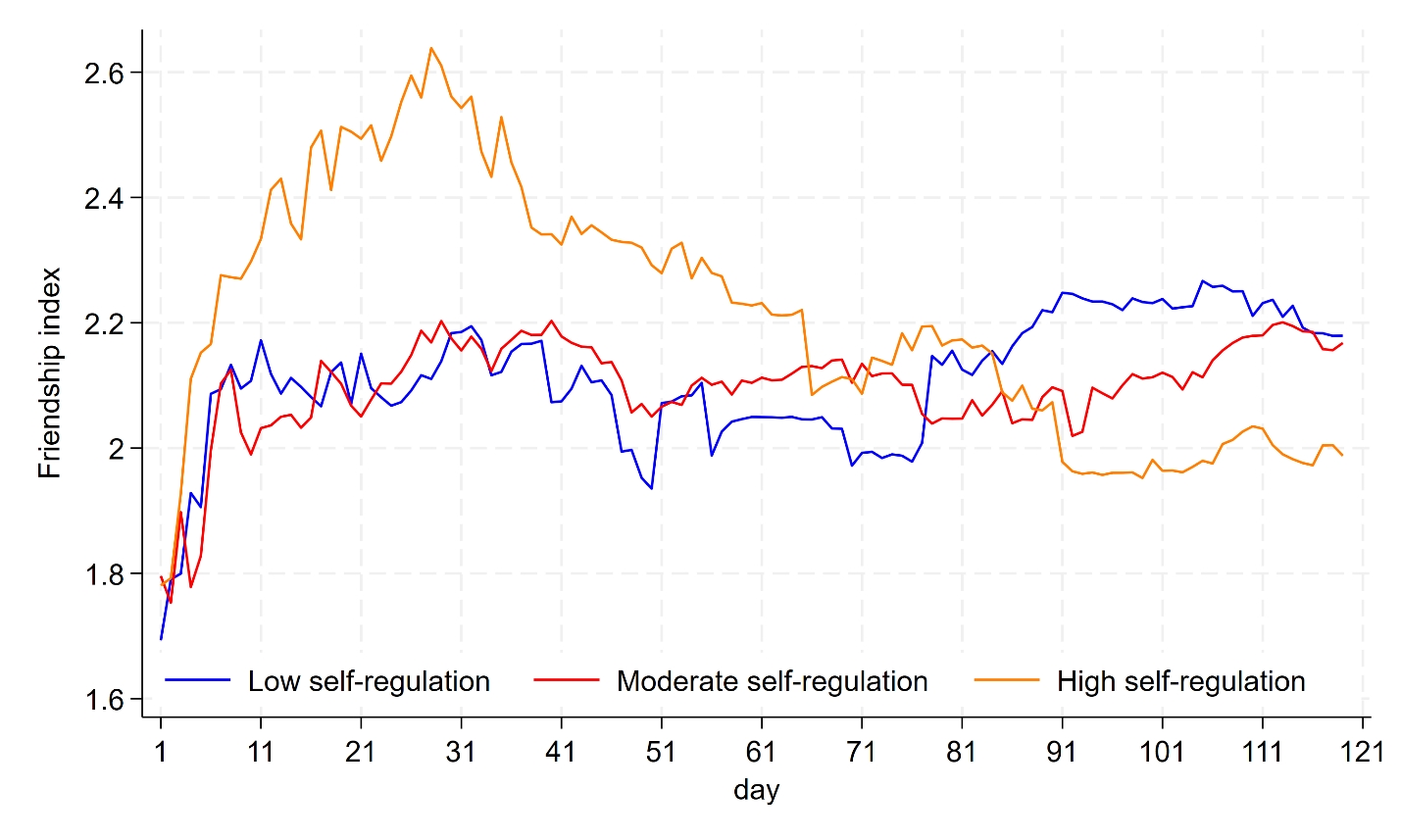


B


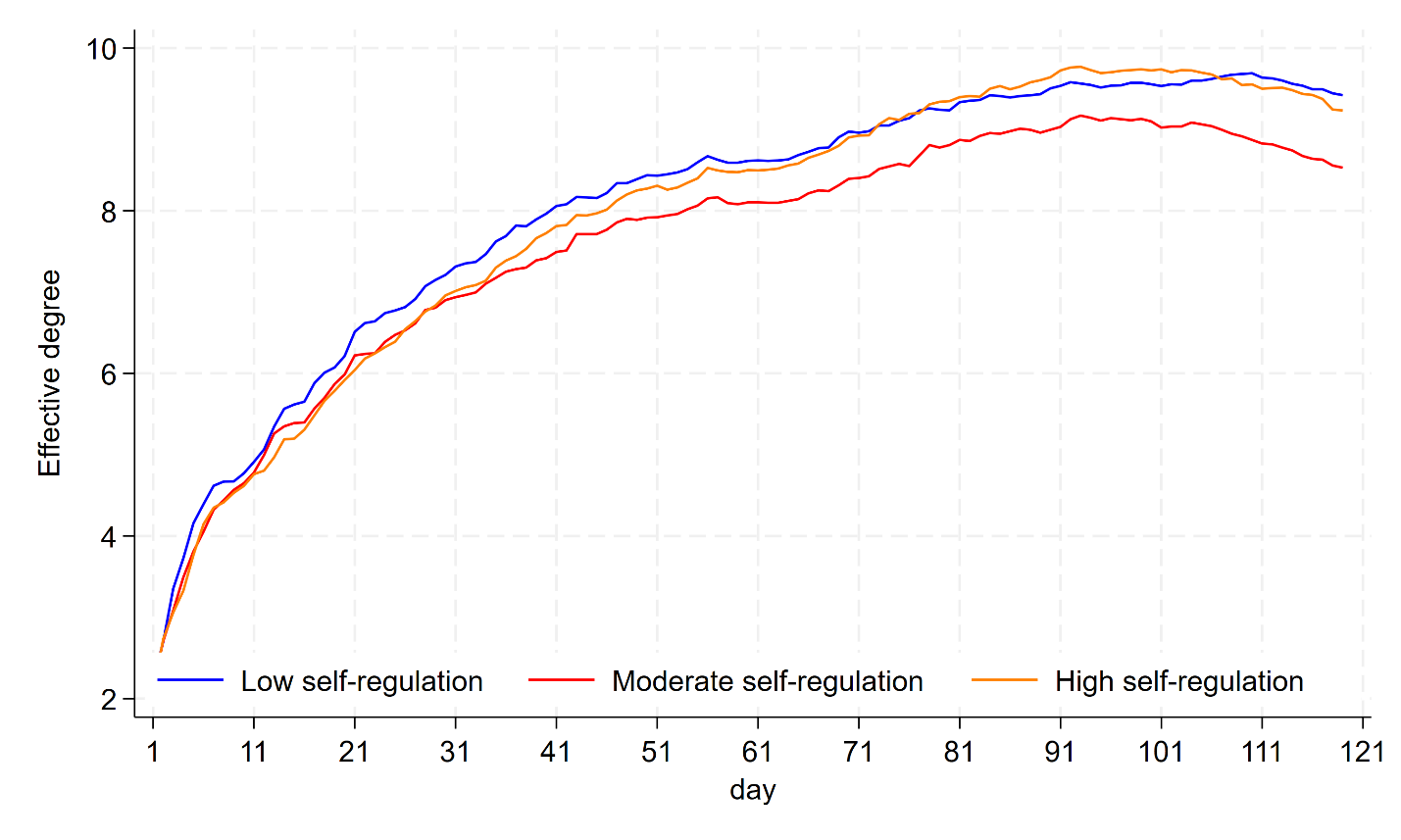


C


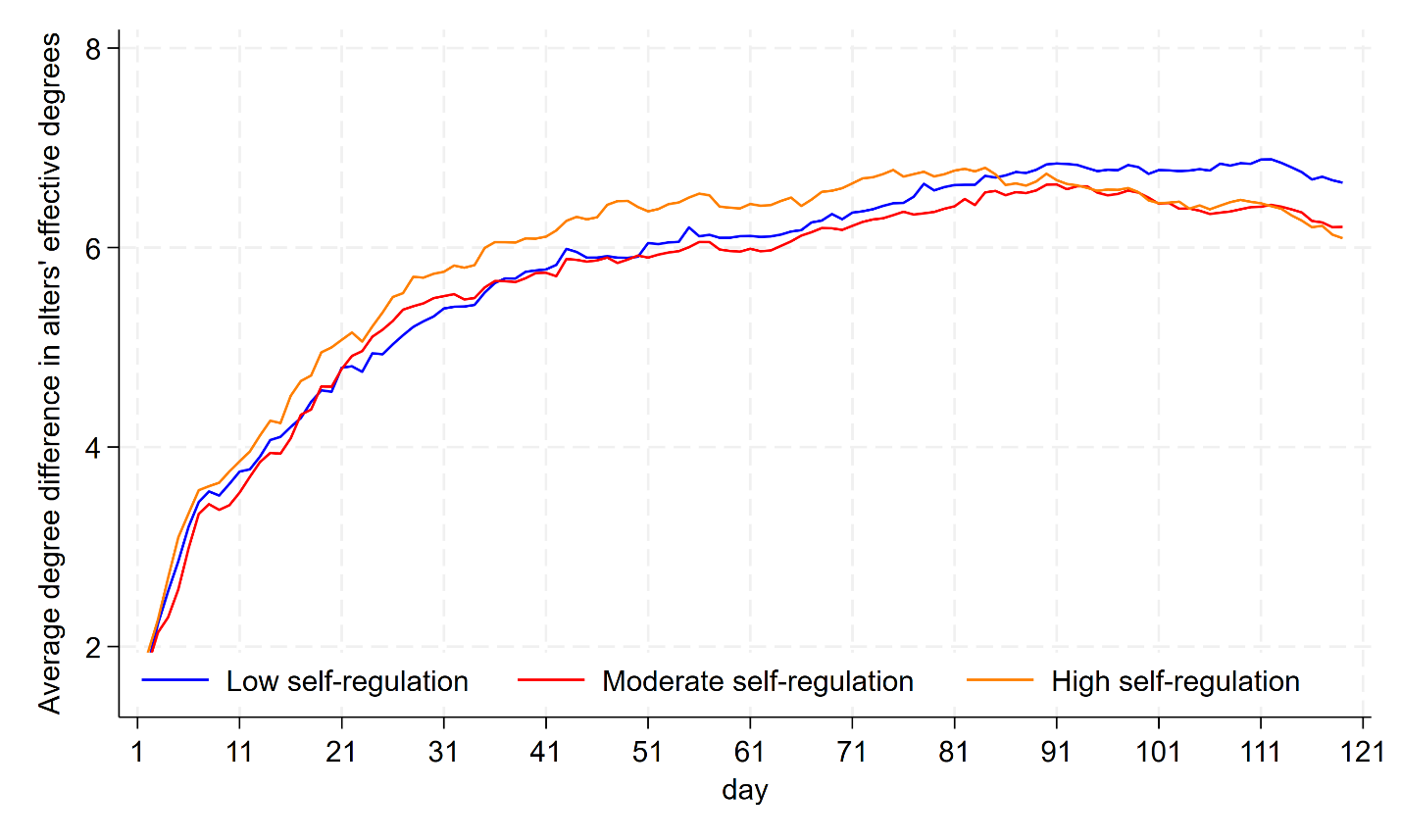


## **Fig. 16** Temporal trend of friendship index, effective degree, and average degree difference in alters’ effective degrees by self-regulation from 08/23/2015 to 12/19/2015

A


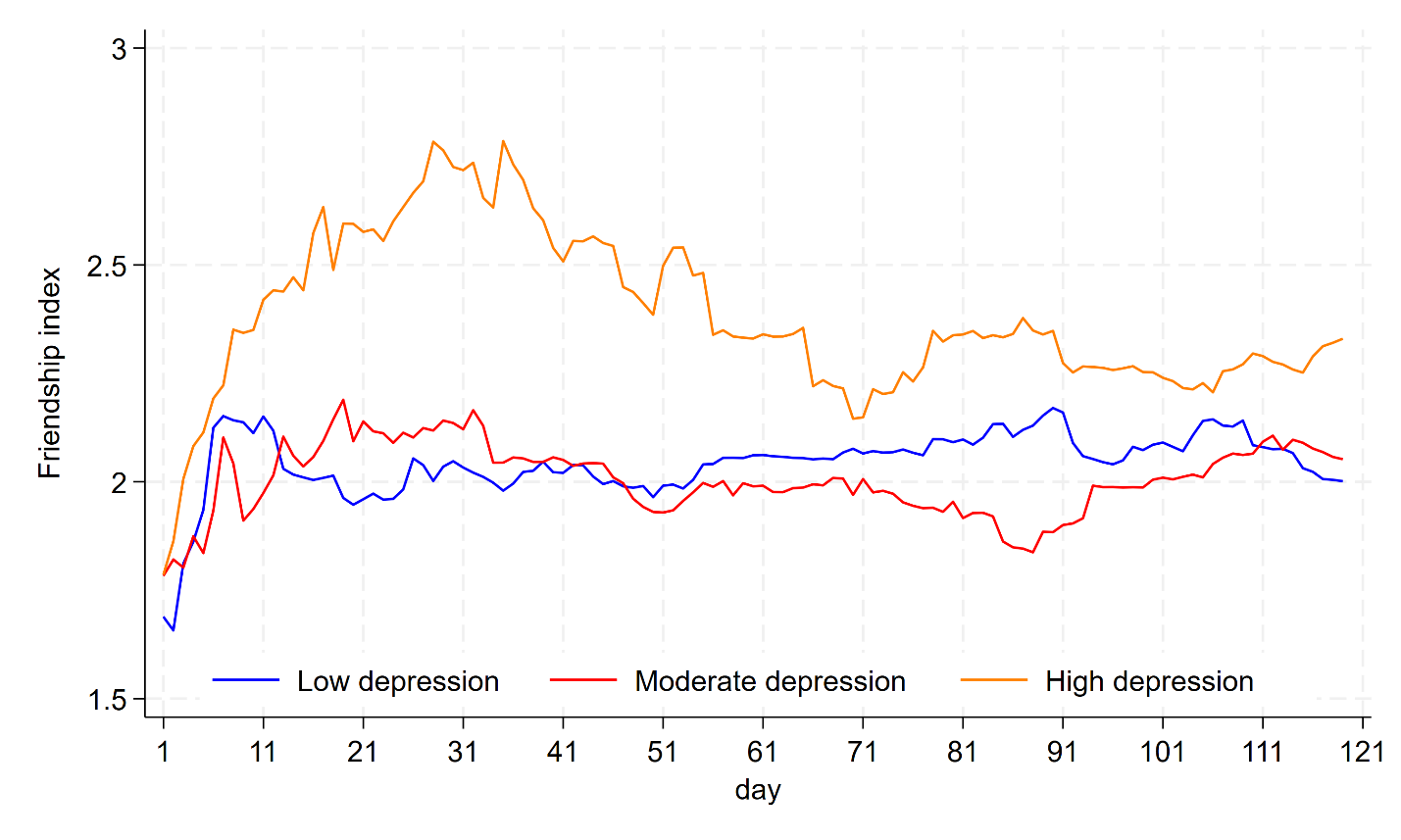


B


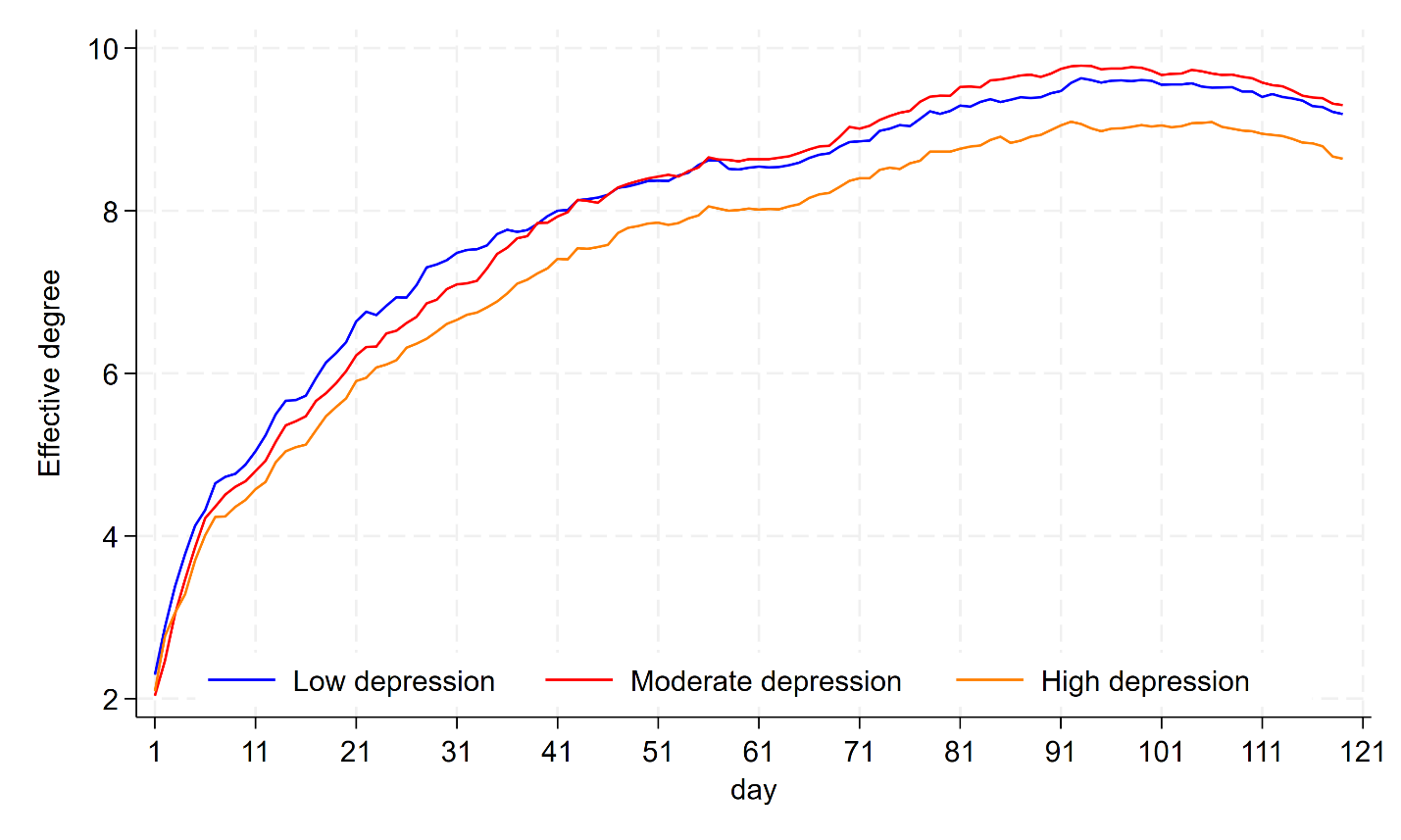


C


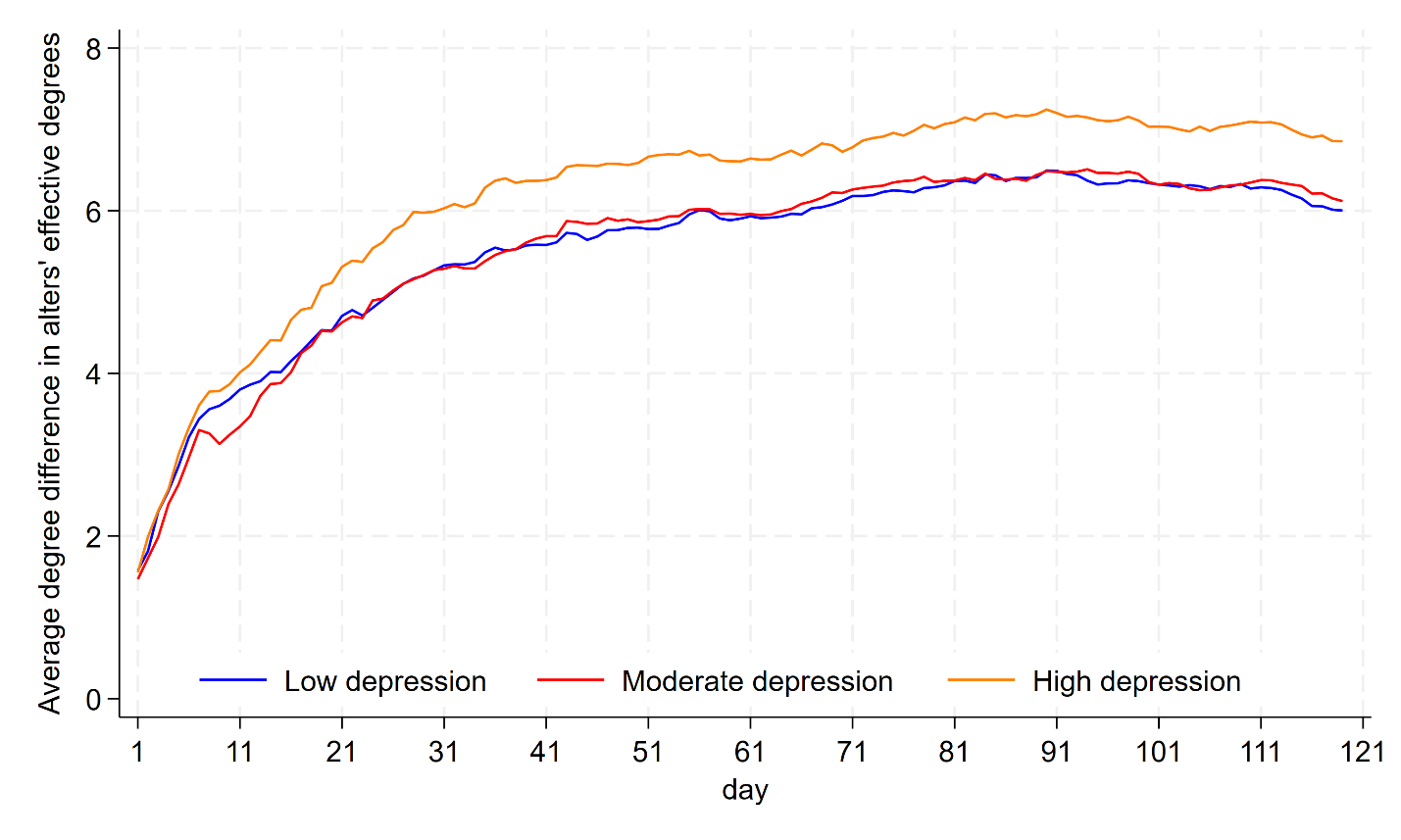


## **Fig. 17** Temporal trend of friendship index, effective degree, and average degree difference in alters’ effective degrees by depression from 08/23/2015 to 12/19/2015
